# Current Diagnosis and Treatment Options for Cutaneous Adnexal Neoplasms with Apocrine and Eccrine Differentiation

**DOI:** 10.3390/ijms22105077

**Published:** 2021-05-11

**Authors:** Iga Płachta, Marcin Kleibert, Anna M. Czarnecka, Mateusz Spałek, Anna Szumera-Ciećkiewicz, Piotr Rutkowski

**Affiliations:** 1 Department of Soft Tissue/Bone Sarcoma and Melanoma, Maria Sklodowska-Curie National Research Institute of Oncology, 02-781 Warsaw, Poland; iplachta@outlook.com (I.P.); marcin.kleibert@interia.pl (M.K.); mateusz@spalek.co (M.S.); piotr.rutkowski@pib-nio.pl (P.R.); 2 Faculty of Medicine, Medical University of Warsaw, 02-091 Warsaw, Poland; 3 Department of Pathology and Laboratory Diagnostics, Maria Sklodowska-Curie National Research Institute of Oncology, 02-781 Warsaw, Poland; szumann@gmail.com; 4 Department of Diagnostic Hematology, Institute of Hematology and Transfusion Medicine, 00-791 Warsaw, Poland

**Keywords:** apocrine, eccrine, neoplasms, tumors, treatment

## Abstract

Adnexal tumors of the skin are a rare group of benign and malignant neoplasms that exhibit morphological differentiation toward one or more of the adnexal epithelium types present in normal skin. Tumors deriving from apocrine or eccrine glands are highly heterogeneous and represent various histological entities. Macroscopic and dermatoscopic features of these tumors are unspecific; therefore, a specialized pathological examination is required to correctly diagnose patients. Limited treatment guidelines of adnexal tumor cases are available; thus, therapy is still challenging. Patients should be referred to high-volume skin cancer centers to receive an appropriate multidisciplinary treatment, affecting their outcome. The purpose of this review is to summarize currently available data on pathogenesis, diagnosis, and treatment approach for apocrine and eccrine tumors.

## 1. Introduction

**Figure 1:**
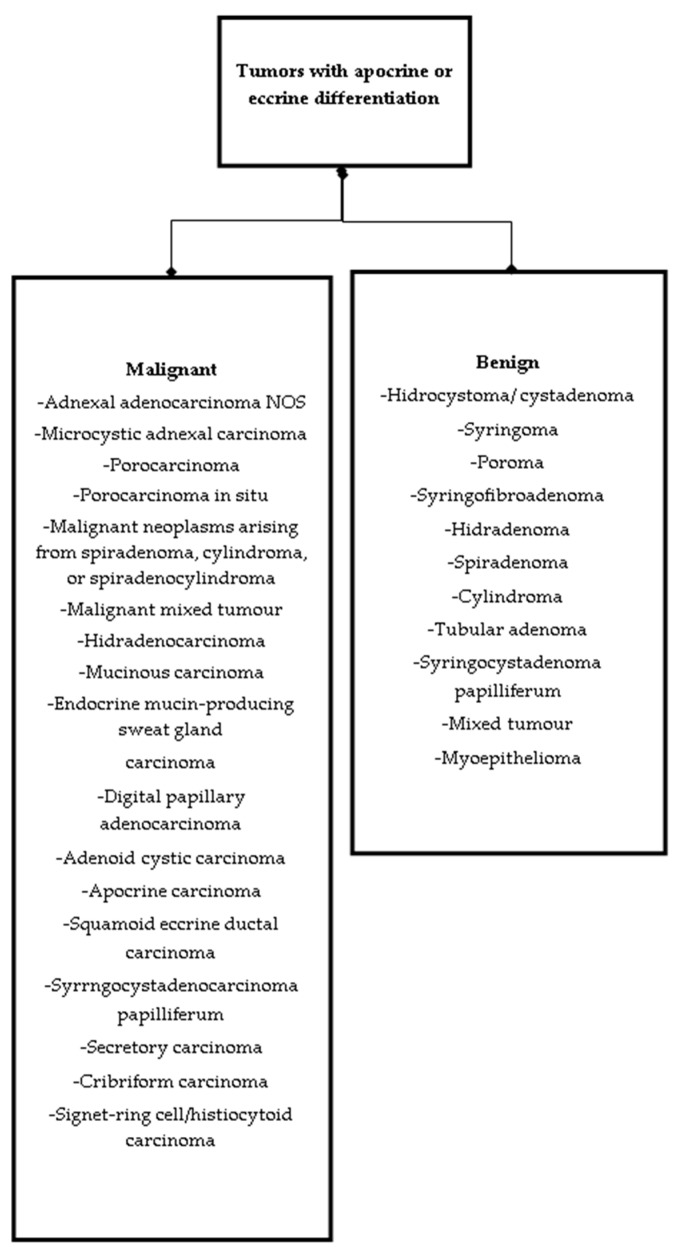
Division of tumors with apocrine and eccrine differentiation.

**Figure 2:**
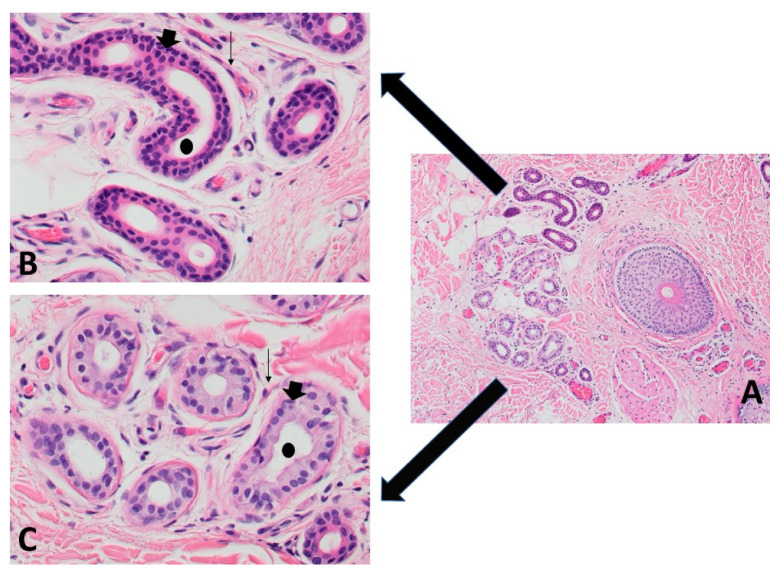
Skin specimen section: (**A**) skin specimen with hair follicle, apocrine and eccrine gland surrounded by connective tissue (40×, HE), (**B**) eccrine glands secrete a fluid directly onto the skin surface, thin arrow—myoepithelial cells, thick arrow—eccrine gland cells, circle—lumen (200×, HE), (**C**) apocrine glands produce a smelling, viscous fluid into the hair follicle, thin arrow—myoepithelial cells, thick arrow—apocrine gland cells, circle—lumen (200×, HE).

Cutaneous adnexal neoplasms are a minor group of skin tumors of follicular, sebaceous, apocrine, or eccrine origin. Tumors developing from sweat glands remain the most heterogeneous subgroup of adnexal neoplasms (Figure 1). Both apocrine and eccrine sweat glands (Figure 2) give rise to benign and malignant tumors. Most often, malignancies may develop from a preexisting benign tumor or arise de novo [1].

**Figure 3:**
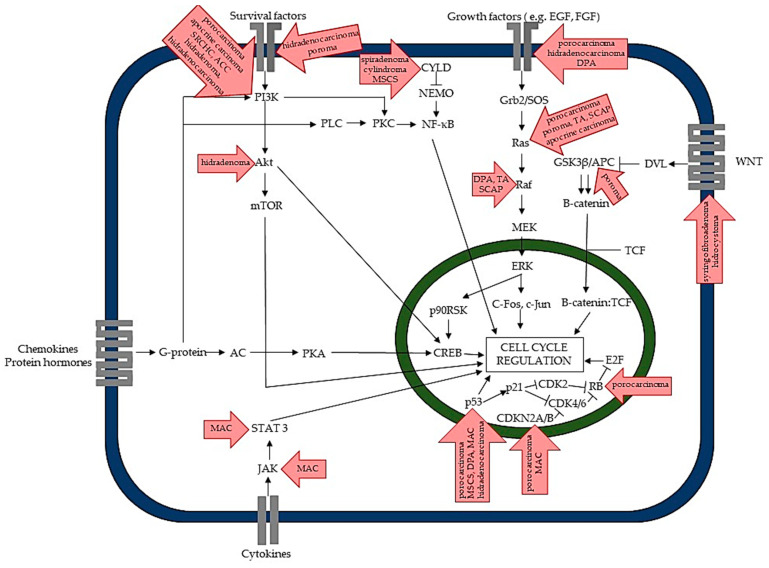
Disturbance of intracellular signaling pathways. Single arrow—activation; double arrow—activation by inhibition; inhibition arc—inhibition. AC—adenylyl cyclase; Akt—protein kinase B; APC—adenomatous polyposis coli; CDK—cyclin-dependent kinase; CDKN—cyclin-dependent kinase inhibitor; CREB—cAMP response element-binding protein; CYLD—ubiquitin carboxyl-terminal hydrolase; DVL—disheveled; ERK—extracellular signal-regulated kinase; EGF—epidermal growth factor; FGF—fibroblast growth factor; Grb—growth factor receptor-bound protein; GSK—glycogen synthase kinase; JAK—Janus kinase; MEK—mitogen-activated protein kinase kinase; mTOR—mechanistic target of rapamycin; NEMO—NF-Kappa-B essential modulator; NF-κB—nuclear factor kappa-light-chain-enhancer of activated B cells; p90RSK—90 kDa ribosomal s6 kinases; PI3K—phosphoinositide 3-kinase; PKA—protein kinase A; PKC—protein kinase C; PLC—phospholipase C; RB—retinoblastoma protein; SOS—Son of Sevenless; STAT3—signal transducer and activator of transcription 3; TCF—transcription factor.

Typically, adnexal neoplasms occur sporadically; however, possible triggers have been reported for a few of them, including UV exposure, radiation, immunosuppression, and antecedent trauma, in the past [2,3,4,5,6,7]. Lesions are often associated with other dermatoses or skin tumors, such as naevus sebaceous for microcystic adnexal carcinoma, tubular adenoma, poroma, and syringocystadenoma papilliferum [8,9,10,11,12,13]. Other neoplasms seem to increase the risk of developing adnexal tumors, and squamoid eccrine ductal carcinoma was observed in patients with chronic lymphocytic leukemia [14]. Lesions such as spiradenoma or cylindroma may develop as a part of Brooke-Spiegler syndrome, a genetic disorder with germline mutations in the *CYLD* (CYLD Lysine 63 Deubiquitinase) gene [15]. Also, multiple syringofibroadenomas on the palms and soles and multiple eyelid hidrocystomas are distinctive for Schöpf-Schulz-Passarge syndrome, the autosomal recessive disease caused by *WNT10A* (Wnt Family Member 10A) gene mutation [16,17]. Other DNA mutations were also reported, including abnormalities commonly found in other cancers, such as *EGFR* (epidermal growth factor receptor), *HRAS* (*HRas* Proto-Oncogene, GTPase), *TP53*, *RB1* (*retinoblastoma protein*), *ATM* (*ATM* Serine/Threonine Kinase), and *PIK3CA* (Phosphatidylinositol-4,5-Bisphosphate 3-Kinase Catalytic Subunit Alpha) (Table 1 and Figure 3) [18,19,20,21,22,23,24,25]. Similar genetic abnormalities were reported in benign and malignant adnexal tumors, and some mutations were found in multiple adnexal neoplasms [26,27,28]. Tumors are occasionally found in patients with several other genetic and systemic disorders, including Down syndrome or diabetes mellitus, Marfan syndrome, Ehler-Danlos syndrome, sarcoidosis, etc., and alopecia areata for syringoma [29,30]. Human papillomavirus (HPV) might also play a role in adnexal tumors’ development, especially for syringofibroadenoma or syringocystadenoma papilliferum [31,32,33,34].

This study aims to analyze cutaneous adnexal tumors for their morphological, histological, or clinical features and discuss possible therapeutic options.

## 2. Tumors with Apocrine and Eccrine Differentiation

### 2.1. Malignant Tumors with Apocrine or Eccrine Differentiation

#### 2.1.1. Adnexal Adenocarcinoma Not Otherwise Specified (NOS)

**Figure 4:**
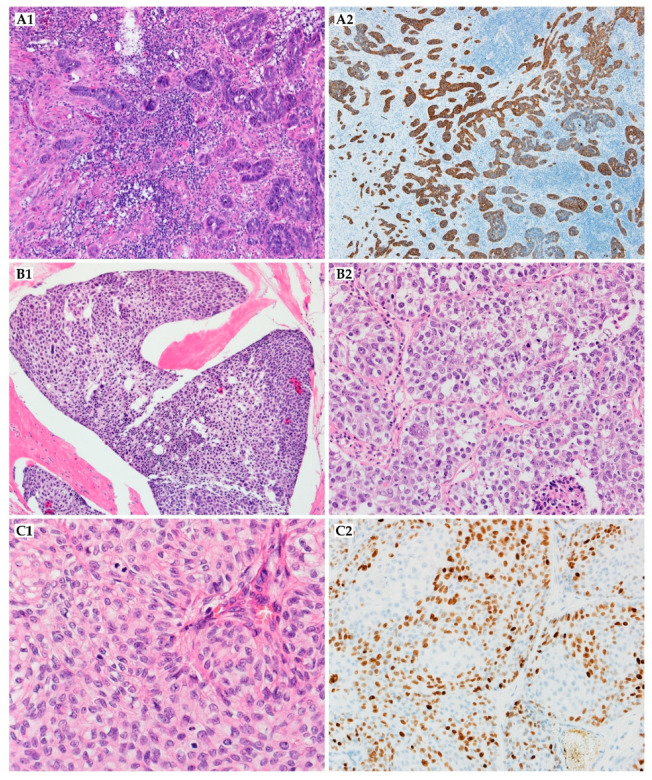
Malignant tumors with apocrine or eccrine differentiation I. (**A1**) (20×, HE) and (**A2**) (20×, CK7): adnexal adenocarcinoma, not otherwise specified, deeply invasive lesion, involves deep dermis and extends to subcutaneous tissues. (**B1**) (40×, HE) and (**B2**): Porocarcinoma characterized by infiltrative borders, and atypical cytological features, increased mitotic activity, and focal necrosis. (**C1**) (400×, HE) and (**C2**) (200×, Ki67): Hidradenocarcinoma, predominantly composed of bland, clear cells, with increased mitotic activity.

Adnexal adenocarcinoma is a primary skin carcinoma with ductal or glandular differentiation, but it lacks other specific histological features for further classification (Figure 4A1,A2) [35]. The pathogenesis is not known. Adults are mainly affected (median age: 72.5 years), with male predominance [36].

Adnexal adenocarcinoma NOS can occur in any skin region, but the most common localization is the head and neck [4]. The primary importance is to rule out the adenocarcinomas metastatic to the skin [37]. There is no consensus on the immunohistochemical biomarker, which is specific for these carcinomas. Adnexal adenocarcinoma NOS can recur and metastasize [35].

No treatment guidelines are available, but multidisciplinary teams (tumor boards) may manage them as porocarcinoma or poorly differentiated/high-risk primary cutaneous squamous cell carcinoma. The preferred treatment is a complete excision (Table 2) [2].

#### 2.1.2. Microcystic Adnexal Carcinoma

Microcystic adnexal carcinoma (MAC), also known as sclerosing sweat duct carcinoma, is a malignant sweat gland neoplasm, usually developing in the head and neck region. MAC risk factors are unknown, although possible triggers include UV exposure, immunosuppression, radiation (in prior history in 19.5–50% of patients), and genetic predisposition [3,8]. Lesions may be associated with multiple syringomas or naevus sebaceous [9,10,38]. MAC is a molecularly heterogeneous neoplasm with inactivated p53 or activated JAK/STAT (Janus kinase/signal transducer and activator of transcription protein family) signaling pathway (Table 1) [39]. A solid variant of MAC, known as solid carcinoma, may be distinguished [40].

Neoplasm usually occurs in older adults of white ethnicity. The mean age is the fifth and sixth decade, although it can occur in any age group, including congenital disease in neonates [18,41,42]. There is a slight female predominance [43]. The yearly incidence is 0.52 cases per 1,000,000 people per year. The most significant group of cases published is 1968 patients in a systemic review [3]. The survival at 1 and 5 years was reported as 98.1% and 88.1%, respectively [44].

MAC presents as an asymptomatic, smooth, non-ulcerated, white or yellowish papule, plaque, or nodule. The lesion typically occurs in the head and neck region [42,45]. Histologically, MAC is typically well-differentiated and deeply penetrating, although it may exhibit subclinical lateral spread. The tumor shows both eccrine and follicular differentiation. Pathologic features occur as keratin cysts or syringomatous ductal differentiation with or without keratin cysts. Additionally, collagenous to scar-like stroma and bland keratinocyte cords are found. Cells show little cytologic atypia and few mitoses. Sebaceous differentiation and invasion of the reticular dermis or nerves are possible. Extension into subcutaneous fat, muscle, or bone has been widely reported. Due to good differentiation, MAC usually does not require to be evaluated by immunohistochemical staining. However, if a tumor cannot be diagnosed based only on histological features, staining with positive markers, including CK17 (cytokeratin 17), CK15 (cytokeratin 15), CK5/6 (cytokeratin 5/6), and p63, should be supportive. Pankeratin, CD5 (cluster of differentiation 5), and CK7 (cytokeratin 7) may be present, although they are not considered specific for MAC [3]. EMA (epithelial membrane antigen) and CEA (carcinoembryonic antigen) expression can highlight ductal differentiation, but GCDFP15 (gross cystic disease fluid protein) and BerEP4 (epithelial cell adhesion molecule/EPCAM) are always negative. The histopathological differential diagnosis includes benign adnexal tumors such as syringoma, desmoplastic trichoepithelioma, and trichoadenoma [6]. Superficial shave biopsies are not recommended for MAC diagnosis because of the lack of possibility to access deep infiltration or perineural invasion, which may lead to misdiagnosis [3].

The ultimate goal is excision with clear (R0) surgical margins. Mohs micrographic surgery (MMS) (overall recurrence rates = 0–12%) or complete peripheral excision (overall recurrence rates = 30–47%) and in-depth margin assessment are recommended as first-line treatment, also in case of the recurring disease. Chiller et al. noticed that a lower recurrence rate was associated with standard surgical excision (1.5% per person-year) than MMS (2.4% per person-year). Instead of this slight difference, MMS has a clear benefit over simple excision in that approximately one-third of tumors treated with simple excision required at least another office visit to clear histological margin, compared to 0% after MMS [46]. Wide local excision (WLE) may be performed, and 2 cm peripheral margins with a minimum deep margin to fascia are suggested because of the high risk of recurrence (up to 30–47%), especially if the surgical margin is 4 mm or less. Topical or superficial destructions (with liquid nitrogen or electrodesiccation and curettage) are not effective treatments because of penetrating growth, at least reaching the reticular dermis. Sentinel lymph node biopsy is not recommended for the staging of MAC. Primary radiotherapy may be considered for nonsurgical candidates. For radiotherapy treatment, a delivered dose is suggested to be greater than 60 Gy (preferably 60–66 Gy) at 2 Gy per fraction. A margin of 3 to 5 cm around the surgical bed is recommended. Adjuvant radiotherapy (60–66 Gy, 2 Gy per fraction, or 45 Gy, 4.5 Gy/fraction) can be considered in case of perineural invasion, considering that occult tumor is persistent at the margins or when margins cannot be achieved surgically. Nodal (2%) or distant (0.2%) metastases are uncommon, and death associated with the disease occurs in 0.1% total count of cases. Nodal radiotherapy for metastatic disease and chemotherapy or immunotherapy for distant metastases are not currently recommended (Table 2), although the case of metastatic MAC with a documented objective response (OR) to chemotherapy (carboplatin and paclitaxel) confirmed by positron emission tomography/computed tomography (PET) was published [3,43,47,48]. Most recently, oral S1 (that contains tegafur (FF) and two types of enzyme inhibitor, 5-chloro-2,4-dihydroxypyridine (CDHP) and potassium oxonate (Oxo) in a molar ratio of 1:0.4:1) was also successfully used for the treatment of MAC on the philtrum [49]. Concurrent chemoradiotherapy was also used for MAC. In the case reported, treatment was delivered over 7 weeks, including 70 Gy of intensity-modulated RT over 35 fractions to the primary site and 4 cycles of concomitant weekly carboplatin (area under the curve 1.5) and paclitaxel (30 mg/m^2^), as carboplatin/paclitaxel have activity and act as a radio-sensitizer in head and neck cancers [50]. A recent single-institution retrospective study based on 53 cases demonstrated that the risk of recurrence increased by 11% per 1 cm^2^ increase in tumor size [51].

#### 2.1.3. Porocarcinoma In Situ and Porocarcinoma

Porocarcinoma, first described by Pinkus and Mehregan in 1963, is an adnexal malignancy originating from the intraepidermal portion of the eccrine or apocrine sweat gland duct. This neoplasm is one of the most aggressive behaviors among all adnexal tumors, compared to other non-melanoma skin cancers. The neoplasm may develop from a benign counterpart, poroma (30–50%); however, porocarcinoma might arise de novo, with some cases reported as a consequence of prolonged radiotherapy [11,52]. DNA sequencing data have shown mutations in *EGFR*, *HRAS*, *TP53*, *RB1*, *ATM*, *ARID1A* (*AT-rich interactive domain-containing protein 1A*), *PIK3CA,* and *CDKN2A* (*cyclin-dependent kinase inhibitor 2A*) [25]. Highly recurrent YAP1-MAML2 (yes-associated protein 1-mastermind-like protein 2) and YAP1-NUTM1 (yes-associated protein 1-NUT family member 1) fusions in most porocarcinomas were found (Table 1) [28]. Porocarcinoma in situ is confined to the epidermis [53].

Eccrine porocarcinoma is estimated to represent 0.005–0.01% of all skin cancers. The largest group of affected patients subsumed into one publication is 453 cases in a meta-analysis [54]. It usually affects people in the sixth up to eighth decades of life; however, it may affect infants and children. Poroma does not appear to have a predilection for race or sex, even though some authors stated that porocarcinoma is more common among male patients [54]. About 10% of all porocarcinomas are in situ lesions [55].

Porocarcinoma is most commonly located on the lower limbs (44%), trunk (24%), or head and neck regions (24%), and other common sites include the face, scalp, and ears (~20%), upper extremities (~11%), abdomen (~9%), and other less significant locations. It may be described as a firm nodule or mass with discoloration of the skin, ranging from erythematous to violaceous. It tends to be more exophytic and ulcerative than a benign one. Its size does not usually reach 2 cm at the time of diagnosis. The lesion’s identification is based on spontaneous bleeding, ulceration, sudden itching, pain, or precipitated growth. It is essential to distinguish porocarcinoma from seborrheic keratosis, pyogenic granuloma, melanotic melanoma, squamous cell carcinoma, basal cell carcinoma, verruca vulgaris, and metastatic adenocarcinoma. At the time of diagnosis, 22.3% patients present with metastatic spread—17% with lymph node metastases, 3.9% with distant metastases, and 1.5% with a locoregional cutaneous spread [54].

**Figure 5:**
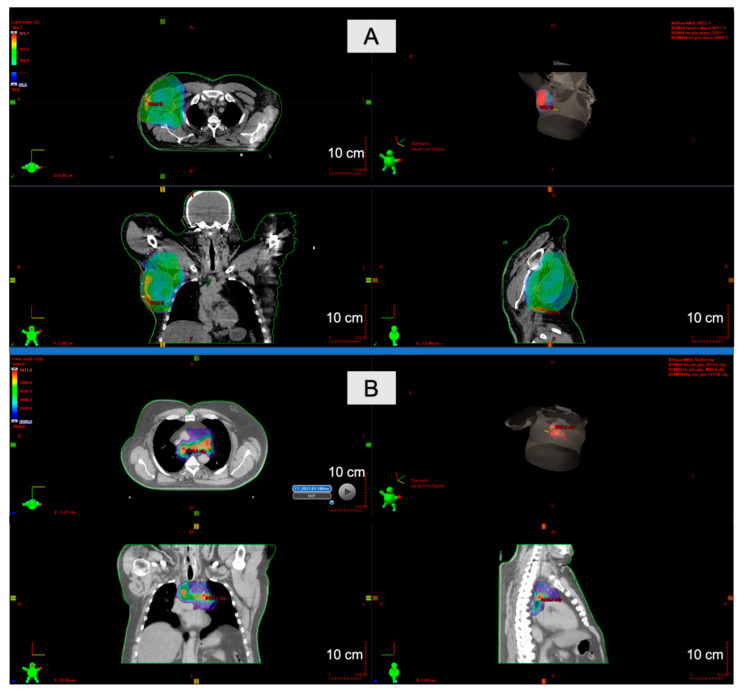
Radiotherapy for eccrine porocarcinoma. The patient received adjuvant radiotherapy (**A**) after axillary lymph node dissection due to locally advanced porocarcinoma with five lymph node metastases with extracapsular extension using intensity-modulated radiotherapy, 60 Gy in 2 Gy fractions. After six months of follow-up, the patient developed lung metastases, and he underwent chemotherapy followed by pulmonary metastasectomy. The first computed tomography after surgery showed mediastinal lymph nodes metastases. Then, the patient received hypofractionated radiotherapy (**B**) for oligometastatic disease using volumetric modulated arc therapy with simultaneous integrated boost, 45 and 30 Gy in 4.5 and 3 Gy fractions.

Porocarcinoma in situ is limited to the epidermis and has a classical poroma pattern with cytological features of malignancy, including nuclear pleomorphism, nuclear hyperchromasia, and atypical mitoses [53]. Porocarcinoma consists of anaplastic cells comprising the epidermis and extending into the dermis (Figure 4B1,B2). The mitotic index is high, and areas of necrosis are frequently seen. In some cases, these cells might be located next to the poroma cells, if not extending from them. CEA and EMA are markers of ductal differentiation. Other epithelial (e.g., CK AE1/AE3) and myoepithelial (e.g., p63, S100) markers may help diagnose, even though the immunohistochemical profile has not been established yet. Porocarcinoma may show various differentiation features, including squamoid tumor cells or melanocytes, Borst Judasson appearance, clear-cell, high infiltrative pattern with hyperchromatic nuclei, and identification of pre-existing poroma or porocarcinoma in situ is highly supportive in the diagnosis [54,56]. The therapeutic approach to porocarcinoma consists of surgery with adjuvant therapy if metastases or/and recurrence occur (Figure 5). Surgery includes WLE, MMS, and amputation. Various excision margins are reported; the most common is 10 mm, although additional are 3, 4, 20, 25, and 30 mm. WLE is the most common therapy. However, local recurrences are reported in approximately 20% of cases, and patients who underwent WLE account for 61% of patients who developed subsequent metastasis. In a recent meta-analysis, local recurrences were reported in 14.7% of patients after WLE and 0.0% after MMS. Similarly, WLE and MMS led to regional recurrences in 25.3% and 0.0%, respectively [57]. More recent cases report that MMS may result in far fewer metastatic cases than excision (0–2.4% after MMS versus 8.8–18% after excision) [56,57]. Lymph node biopsy should be considered if patients possess high-risk features, including lymphovascular invasion, high proliferative index (>14 mitoses in 10 high-power fields), increased tumor depth (>7 mm), poorly differentiated neoplasm, and location of the primary tumor (head/neck region appears to be responsible for a comparatively smaller proportion of lymph node metastases than other primary locations) [56]. The biopsy may successfully identify occult lymph node metastasis (~50% of patients) in patients without palpable lymphadenopathy. If lymphadenopathy is present, lymphadenectomy is performed. However, prophylactic lymph node resection does not appear to improve disease-free survival. Adjuvant therapy includes chemotherapy (5-fluorouracil, cisplatin, doxorubicin, docetaxel, vincristine, cyclophosphamide, carboplatin with epirubicin, paclitaxel, bleomycin, and mitomycin), radiotherapy, chemoradiotherapy, and sporadically, interferon or phototherapy. However, great responses are not usually seen in the reported cases, and only in a few of them was a beneficial effect obtained, for example, the combination of chemotherapy (cisplatin/docetaxel) and radiation (50 Gy/25 fractions) in a patient with metastatic disease resulted in complete response [11,58,59]. De Bree et al. successfully applied extensive cutaneous metastases with topical 5-fluorouracil combined with intraarterial docetaxel [60]. Complete rehabilitation in a case with multiple skin metastases under hyperthermic perfusion with the administration of melphalan and intra-arterial 5-fluorouracil was reported by Briscoe et al. [61]. In a case of Aaribi and colleagues, a complete response was obtained after three cycles of docetaxel [62]. Fujimura et al. presented with cyberknife radiosurgery as a novel therapeutic option for inoperable metastatic eccrine porocarcinoma [63]. However, in a recent meta-analysis, adjuvant treatment did not significantly improve the prognosis (*p* = 0.458), nor the overall survival (*p* = 0.790) [57]. Moreover, it can be observed that porocarcinoma is a very aggressive neoplasm and patients die a few months after the diagnosis, regardless of the type of management. The death rate is 67% among patients with regional lymph nodes involvement, and the mean time from initial presentation to treatment initiation is 8.5 years (Table 2) [54,57].

#### 2.1.4. Malignant Neoplasms Arising from Spiradenoma, Cylindroma, or Spiradenocylindroma

Malignant neoplasms arising from spiradenoma, cylindroma, or spiradenocylindroma (MSCS) are rare sweat gland carcinomas whose diagnosis is based on recognizing a pre-existing benign one of lesions [64]. There is a lack of information about pathogenesis. In some cases, the mutations in *TP53* can be found [65]. If the lesion is a part of Brooke-Spiegler syndrome, which manifests as numerous adnexal lesions (mostly benign) as spiradenoma, cylindroma, spiradenocylindroma, and trichoepithelioma, the CYLD mutation is present (Table 1) [64,66]. It is proposed that sun exposure or immunosuppression may contribute to the lesion’s evolution [67]. Malignant neoplasms arising from spiradenoma, cylindroma, or spiradenocylindroma usually affect people in the sixth decade of life (median age: 63.5 years), with no significant sex bias [64,68]. The most remarkable group, which was described, included only 24 cases for neoplasms arising from spiradenoma, cylindroma, or spiradenocylindroma, and 72 cases for malignant spiradenoma [64,69].

Patients usually present with a solitary nodule on the head or neck. The mean size of the lesion is 4 cm (size range, 2.2 to 17.5 cm). This neoplasm can occur as the sporadic or the part of Brooke-Spiegler syndrome, and the microscopic presentation of the malignant component is quite variable. According to the WHO classification, we can distinguish such subtypes as the basal cell adenocarcinoma-like, low-grade (BCAC-LG) pattern, the basal cell adenocarcinoma-like, high-grade (BCAC-HG) pattern, the invasive adenocarcinoma NOS, and the sarcomatoid (metaplastic) carcinoma [66]. The differences between each subtype of this tumor are based on size, pleomorphism, mitotic activity (and the presence of the atypical and necrotic cells), infiltration of stroma, and composition (the sarcomatoid carcinoma is composed of the epithelial and metaplastic cells). The immunohistochemical staining is very useful in diagnosis. In general, the tumors with ductal differentiation are positive for EMA and CEA. Additionally, MYB expression is lost in the malignant component [66].

In a microscopic view, low-grade tumors can be easily mistaken for benign neoplasm. The features typical for malignant transformation include loss of the dual cell population, cytological atypia, increased mitotic activity, and loss of intra-tumoral lymphocytes. They should be carefully examined to distinguish between malignant and benign lesions. Neither disease-related deaths nor local recurrences were found in BCAC-LG in the series of Granter et al. [70]. On the contrary, sarcomatoid and metaplastic variants seem to present a favorable prognosis.

A recent meta-analysis of reported cases of cutaneous sarcomatoid carcinomas revealed a 25% 5-year disease-free survival rate [71]. Metastatic disease and mortality have only been reported with morphologically high-grade tumors. The first-line treatment is WLE because of the high recurrence rates and the potential to metastasize. In 35 patients without metastatic spread, a disease-free survival rate of 100% at 33 months after WLE was obtained [69]. Laser ablation, MMS, cryotherapy, retinoic acid, trichloroacetic acid, carbon dioxide laser, and radiotherapy can be performed, but only as additional treatment [72,73,74,75,76]. In extensive lesions, resurfacing with split skin grafts is the method of choice for covering the defects (Table 2) [77].

#### 2.1.5. Malignant Mixed Tumor

A malignant mixed tumor of the skin, also known as malignant chondroid syringoma, is usually derived from apocrine glands. Genetic abnormalities may be involved in pathogenesis; however, currently, only one case with a fusion of the genes *PHF1* (PHD finger protein 1) and *TFE3* (transcription factor E3), commonly found in other neoplasms, has been reported (Table 1) [78].

Less than 50 cases of malignant mixed tumor have been described in the literature [78]. Neoplasm occurs mainly in the elderly, slightly more frequent in the fourth and seventh decades, but it can occur at any age. Additionally, it is twice more common in women than in men [79].

Patients present with firm, circumscribed, asymmetrical solitary nodule or tumor (diameter from 2 to 15 cm) without any specific features, sometimes rapidly growing. Malignant variants seem to have a predilection for the trunk and extremities, whereas distant metastases are usually found in the lungs, bone, and brain [78].

Diagnosis of the malignant mixed tumor should only be made when a pre-existing benign mixed tumor is identifiable. It consists of both epithelial and mesenchymal tissue, usually as a benign tumor with malignant components. The malignant epithelial component is composed of neoplastic cells with the invasion of surrounding tissue, hyperchromatic nuclei, high mitotic index, and occasional necrosis areas. It may show an adenocarcinoma (apocrine), myoepithelial carcinoma, carcinoma NOS, or sarcomatoid (metaplastic) pattern. The remnant mesenchymal component may exhibit myxoid, chondroid, osteoid, adipose, or fibrous features surrounding clusters of epithelial cells [78]. Immunohistochemical stains are often positive for CK5/6, CK7, p63, EMA, CEA, and S100, but not specific [80]. Malign mixed tumors have to be taken into account in the differential diagnosis of epithelioid tumors with myxoid stroma arising in deep soft tissue.

WLE with clear margins is the optimal therapeutic approach to the malignant mixed tumor. However, the clinical course is unpredictable. All patients with adequate long-term follow-up may develop recurrence or metastasis [81]. Half of the reported cases had local recurrences (on average after 23 months). In contrast, nodal and distant metastases were observed in 39–42% (on average after 50 months) and 36–40% (on average after 66 months) of the cases, respectively. Of these, the death rate was 23% [78]. Adjuvant radiotherapy, with or without chemotherapy, has shown limited contributions [80]. In some cases, chemoradiotherapy combined with operation, including radiotherapy (44 Gy before operation and 50 Gy after it, 2 Gy/fraction) and cisplatin, 5-fluorouracil, irinotecan, and taxotere, can be useful. In the malignant cutaneous mixed tumor with pulmonary metastases, the patient showed a favorable response to high-dose-rate (HDR) endobronchial brachytherapy [82]. In some cases, metastases or local recurrence may be found more than ten years after initial resection [19,80]. Due to the lesion’s long-term potential to recur and metastasize, regular follow-up and examination of the patient are recommended (Table 2).

#### 2.1.6. Hidradenocarcinoma

There are no established mutations that can condition hidradenocarcinoma formation. Mutations in *MALM2*, p53, and Her2/neu (receptor tyrosine-protein kinase erbB-2) can play a role in cancerogenesis, but more analysis is necessary. The minority of lesions present translocation (11;19), which involves the genes CRTC1 (CREB-regulated transcription coactivator 1) and MAML2. Additionally, less than one-fifth of hidradenocarcinomas have a mutation in *TP53* (Table 1) [83,84]. It usually develops de novo, not with hidradenoma [85].

Hidradenocarcinoma is a rare malignant adnexal tumor originating from sweat glands, which accounts for less than 6% of malignant eccrine and 0.001% of all tumors. The range between 55 and 70 years old is the most common age group presentation of hidradenocarcinoma (mean of onset is fifty). Hidradenocarcinoma presents with a slight female predominance. It can develop de novo and rarely results from a preexisting hidradenoma. Radiation exposure in history has been reported in some cases [86]. Hidradenocarcinomas typically present as asymptomatic, well-circumscribed nodules over the superficial skin, but some patients may feel discomfort, pain, or have ulceration and bleeding upon physical contact. It typically occurs on the head and neck and rarely on the extremities. At some point in time, the tumor may present an aggressive clinical course with regional or distant metastases, usually to the lymph nodes, but even in this phase of the disease, the patient may be asymptomatic. This carcinoma consists of different cell types, including clear, squamoid, oncocytic, or mucinous cells, and transitional elements. The neoplastic cells exhibit atypical mitotic figures and nuclear pleomorphism (Figure 4C1,C2) [87]. It has a diverse immunohistochemical profile, and overexpression of HER2 was described. AR (androgen receptor), PR (progesterone receptor), ER (estrogen receptor), HER-2, and EGFR expression were present in 36%, 16%, 27%, 12%, and 85% in immunohistochemical staining. Additionally, trisomy or polysomy of *EGFR* was detected in 30% of cases, and mutations of *PIK3CA*, *AKT-1*, and *TP53* genes were detected in 23% of patients [86].

The gold standard is excision with at least a 3 cm margin, which should be taken for hidradenocarcinoma. Still, if these large margins cannot be respected because of anatomical or functional conditions, the pathologist’s strict histological examination of lateral margins is needed. Approximately 67% of patients develop metastasis mainly to regional lymph nodes (~39%), and distant metastasis (~28%) includes bone, lung, skin, pleura, and other visceral organs [86].

Local recurrence rates following surgery range from 10% to 50%. Some authors did not notice any impact of adjuvant chemotherapy and radiotherapy on local control or survival [88]. Radiation therapy may be given in the presence of local recurrence factors or after the operation as adjuvant therapy (positive margins, recurrent tumors, and positive lymph nodes when further surgery is not possible). High doses ranging from 45 to 70 Gy are recommended [47,89].

The recommended first-line chemotherapy consists of a 5-fluorouracil-based regimen and capecitabine, and second-line agents included doxorubicin, platins, cyclophosphamide, vincristine, and bleomycin [87]. Depending on immunohistochemical and genetic analysis, the targeted therapy can be administered (trastuzumab, tamoxifen, PI3K/Akt/mTOR pathway inhibitors) when the expression of the receptors is on the correct level. These treatment methods may play a role in stabilizing the disease, but further studies are required [90]. The five-year postsurgical survival rate is less than 30% (Table 2) [86].

#### 2.1.7. Mucinous Carcinoma

Mucinous carcinoma (MC), also known as colloid carcinoma or gelatinous carcinoma, is a rare low-grade malignant neoplasm, first described by Lennox et al. in 1952 [91]. It was believed to be of eccrine origin; however, this neoplasm demonstrates apocrine-type differentiation [92]. It is more widely postulated that endocrine mucin-producing sweat gland carcinoma may be a precursor to MC, despite a different MYC expression [93].

The median age at diagnosis is the seventh and eighth decades. Data standardized for specific populations show that there is no sex predominance, nor an increased prevalence of white patients [94]. Furthermore, Asian patients seem to have better outcomes compared to white patients [92]. A total of 411 cases were included in a review, and it is the biggest group of patients subsumed into a publication. The age-adjusted incidence was 0.024 tumors per 100,000 person-years and did not significantly differ between men and women [94].

The lesion usually presents as slow-growing, erythematous, asymptomatic nodules measuring 0.5 to 7 cm in diameter, although larger variants have been reported. Patients with larger initial tumors (>1.5 cm) have increased rates of recurrence and metastasis. It is usually translucent, reddish, or grey-blue [95]. The lesion is typically found in the head and neck region (86.9%), frequently on the eyelid (38.7%), or in the brow region. Eyelid tumors seem to be more likely to present with the distant disease (15.2%) than other head and neck locations [94]. Tumors located on the trunk (9.7%) compared to those on the head and neck may be predictive for the bad outcome (odds ratio (OR) = 103.24; *p* = 0.005) [92]. The characteristic dermoscopic findings are translucent gray globules or area, linear irregular vascular pattern, and whitish structure [96].

Histologically, primary cutaneous MC appears similar to other mucinous carcinomas, showing a lobular architecture with solid, cystic, cribriform, papillary, or mixed growth patterns. Lesions exhibit tumor nests suspended in abundant mucin pools with focal strands or nests of neoplastic cells infiltrating the dermis. Cells are characterized by bland cytological features and occasional mitoses [97]. Neuroendocrine differentiation and immunophenotype can be identified locally. Immunohistochemical profile of MC includes positivity for CK7, CAM5.2, EMA, CEA, GCDFP-15 (gross cystic disease fluid protein 15), RCC (renal cell carcinoma) antigen, WT1 (Wilms’ tumor protein), ER, PR, and AR [93,98,99]. Myoepithelial markers’ staining (p63, calponin) may also be positive. CK7 positivity and CK20 negativity may be useful in distinguishing cutaneous metastases of gastrointestinal MC from primary cutaneous neoplasm. Extensive, clinical exclusion of cutaneous metastasis of other adenocarcinoma lesions (breast, colon, prostate, lung, salivary glands, ovaries, pancreas, and kidney) is recommended before making a diagnosis of primary cutaneous MC [94].

The differential diagnosis for mucinous carcinoma clinically would also include other benign and malignant eccrine tumors, sebaceous carcinoma, cystic basal or squamous cell carcinoma, epidermoid cyst, pyogenic granuloma, hemangioma, Kaposi’s sarcoma, neuroma, or pilomatrixoma [100].

Morbidity associated with MC is mainly related to incomplete excision. The therapeutic approach includes standard surgical resection and WLE. The majority of patients are treated with traditional excision, although immunoperoxidase-guided MMS is performed in some cases, which may increase sensitivity for detection of mucinous carcinoma and help to perform complete tumor removal. In a meta-analysis of 159 cases, 136 patients underwent traditional surgical excision and 34% of them recurred of metastasized, whereas only 13% of 15 cases treated with MMS recurred and no metastatic spread was reported. Mucinous carcinoma has an indolent course and a good prognosis, although late recurrences and rare metastases (5.8–6.1%) have been reported, especially in cases with larger initial tumors (>1.5 cm). Interestingly, the Asian race was a good predictive factor for better outcomes than white (OR = 0.02; *p* = 0.01), as well as older age compared to younger (OR = 0.93; *p* = 0.04) [92,94]. Thus, it is recommended that a wider margin of at least 10 mm should be excised, especially if an intraoperative frozen section facility is not available. Although adjuvant hormone treatment with the anti-estrogenic drug, including tamoxifen, was suggested to decrease recurrence, no remarkable effect was obtained [98]. MC, especially recurrent tumors, is usually resistant to chemotherapy and radiotherapy (Table 2) [97].

#### 2.1.8. Endocrine Mucin-Producing Sweat Gland Carcinoma

Endocrine mucin-producing sweat gland carcinoma (EMPSGC) is a low-grade primary cutaneous neoplasm. It was proposed that EMPSGC is an in situ precursor of primary mucinous adenocarcinoma with neuroendocrine features [93]. However, the more favorable prognosis of neuroendocrine-type mucinous sweat gland adenocarcinoma with reduced recurrence and absence of metastasis compared to other types has been propounded. In a recent case series, the ratio of EMPSGC with invasive mucinous component was 33.3% [101].

Neoplasm typically occurs in women in the seventh decade of life, ranging from 47 to 87 years. About 100 cases of EMPSGC have been reported, and 63 patients are the most significant series of cases included in a publication [101].

The lesion, usually found on the periorbital skin and the eyelid (~66%), is frequently skin-colored, slowly growing nodule, cyst, or swelling with associated erythema, telangiectasia, and madarosis [102]. Dermatoscopic features include pink aggregations to reddish globules (pink ovoid nests) that show a cobblestone appearance, and each globule is separated with white to pink meshes of bands [103].

Histologically, the tumor is well-circumscribed, with partially cystic and partially solid, papillary, or cribriform architecture. Cysts are filled with solid proliferations palisading around fibrovascular cores. Neoplastic cells are epithelial cells, resembling bland eccrine ductal cells, with bluish cytoplasm and stippled, “salt and pepper” chromatin. Both intracellular and extracellular mucin may be present. A low mitotic index is reported. Additional features include rosette-like structures, fibrous stroma, pigment incontinence, and reactive changes in the overlying eyelid epidermis. Due to similar morphologic and immunohistochemical features, it is called the cutaneous analog of the breast solid papillary carcinoma. Tumors might be composed of in situ and invasive mucinous components. These lesions should be diagnosed as mucinous adenocarcinoma. Progression might be signified by pools of extracellular stromal mucin and/or infiltrating tumor glands and nests that lacked an intact myoepithelial rim. Immunohistochemical findings include CK5/6, CK8 and CK18, CAM5.2, WT1, GCDFP15, ER, PR, and variable expression of synaptophysin, chromogranin, and neuron-specific enolase [99,101,102,104,105,106,107]. Myoepithelial markers such as calponin, actin, p63, and S100 might help to detect myoepithelial cells [106,108].

The therapeutic approach includes surgical resection, typically as wedge excision or MMS. It seems to be an appropriate intervention to diminish the risk of progression to invasive carcinoma or recurrence (recurrence rate ~9.5% after excision) [101]. Only one case with locoregional metastasis and another with systemic spread has been reported; therefore, no metastatic disease guidelines have been developed (Table 2) [109,110]. Adjuvant radiotherapy was administered in one case with histologic evidence of vascular and perineural invasion (60 Gy, 2 Gy/fraction). The patient was followed every 3 months, with repeat imaging every 6 months without evidence of recurrence after 1.5 years [111].

#### 2.1.9. Digital Papillary Adenocarcinoma

Digital papillary adenocarcinoma (DPA) is a rare adnexal malignancy. Due to the lack of association between histopathologic features, such as poor glandular differentiation, cellular atypia, necrosis, blood vessel invasion, tumor recurrence, metastasis, or overall outcome, all papillary adenocarcinoma should be considered malignant and necessitate intervention [112]. Several genetic alterations have been reported, including mutation of suppressor gene *TP53*, somatic *BRAF-V600E* (serine/threonine-protein kinase B-Raf) mutation, and overexpression of FGFR2 (fibroblast growth factor receptor 2) (Table 1) [20,21,22]. Etiology is unknown; however, a history of antecedent trauma has been reported in a few cases [7].

Neoplasm is most frequent in Caucasian men. Patients are usually in the sixth and seventh decades of life, although a few cases of tumors occurring in patients below the age of 20 years have been reported [113]. The overall incidence is estimated to be 0.08 per 1,000,000 person-years. The largest group of patients included in a publication is 94 cases [22,112,114].

**Figure 6:**
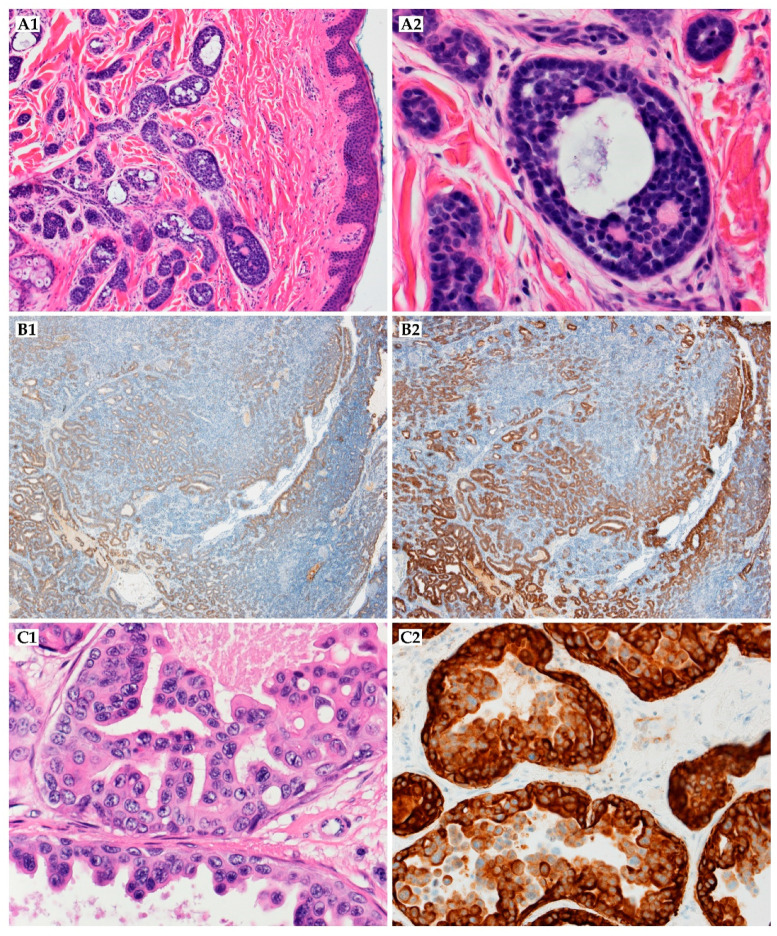
Malignant tumors with apocrine or eccrine differentiation II. (**A1**) (20×, HE) and (**A2**) (400×, HE): Adenoid cystic carcinoma, the coexistence of small bi-layered ducts and pseudocysts. (**B1**) (20×, S100) and (**B2**) (20×, CK7): Digital papillary adenocarcinoma with multinodular growth, cystic, papillary, and glandular structures showing diffuse expression of S100 (in both luminal and myoepithelial cells) and CK7 (highlights the luminal borders). (**C1**) (400×, HE) and (**C2**) (400×, CK5/6): Apocrine carcinoma, neoplastic cells with abundant eosinophilic cytoplasm with a strong and diffuse expression of CK5/6 which favors primary skin lesion.

Lesion mostly presents as a slow-growing, painless nodule located on the volar surface of the digit near the distal interphalangeal joint or in the periungual region. However, other sites, such as the face, may be possible. The nodule may vary in color, from skin-colored to blue-gray. Tumors range in size, from 3 to 50 mm [112,115]. Other skin lesions such as a ganglion cyst, foreign body or pyogenic granuloma, paronychia, glomus tumor, squamous cell carcinoma, hemangioma, giant cell tumor, osteomyelitis, and soft tissue infections should be taken into account in the differential diagnosis. Metastases are usually found in the lungs, followed by lymph nodes, brain, skin, bones, and kidneys, which can develop even after 20 years following initial treatment [113,116]. Histopathologically, it shows solid, cystic, and papillary structures consisting of basaloid tumor cells. Cystic spaces may be filled with eosinophilic lacquered material. Hyalinized stroma is noted in some cases, rarely calcified. Cytologic atypia is mild to severe, with an elevated mitotic index [45]. Necrosis with inflammatory cells may be observed. Focal keratinization, vascular dilatation, and clear cell change have been reported [113]. The immunohistochemical feature is that all tubular structures stain with CK7 (Figure 6B2). They are surrounded by a positive actin layer of myoepithelial cells, which may also be highlighted with calponin, podoplanin, or p63 [22,113]. Other positive stains include S100 (Figure 6B1), pan-cytokeratin, PHLDA1 (Pleckstrin homology-like domain family A member 1), and CAM 5.2 [115,117]. There are no histological features that are useful in metastasis and recurrence prediction. WLE without routine sentinel lymph node biopsy appears to be a widely acceptable first-line treatment. When tumors do not have an aggressive nature, complete excision is sufficient despite the malignant nature. However, recurrence or metastases are possible, even after performing amputation or re-excision, required in 34% of patients after initial excision. In some reported cases, MMS has been successfully used [118,119]. Excision with or without sentinel lymph node biopsy may result in similar outcomes [120]. Recurrence rates range from 16% to 50% and can be reduced to 5% after excision with a clear resection of the margins (R0), and metastases are found in 14% to 41% of cases [112]. The role of adjuvant therapies is unknown; however, complete regression of the DPA after radiotherapy has been reported [112,116,117]. There are no approved therapeutic approaches for metastatic disease, and neither systemic treatment nor radiotherapy are recommended (Table 2) [22]. The follow-up should be long-term because of insidious growth and the high recurrence rate [7].

#### 2.1.10. Adenoid Cystic Carcinoma

Adenoid cystic carcinoma (ACC) is a rare malignancy, histologically identical to adenoid cystic carcinomas found in other sites, including salivary and lacrimal glands, breast, vulva, uterine cervix, external auditory canal, trachea, and bronchi [121,122]. Primary cutaneous ACC, first described by Boggio in 1975, has a more indolent course than its salivary counterpart [123,124]. Its origin has not been determined yet, though some observational evidence suggests that ACC may derive from apocrine glands, including ceruminous glands of the external ear canal [125]. ACC has been reported following treatment for childhood acute lymphoblastic leukemia, rheumatoid arthritis, and Hashimoto’s thyroiditis, with other lymphoproliferative, immunologic, or immunocompromising conditions, which in few cases preceded ACC [5]. MYB protein expression corresponding to *MYB-NFIB* (nuclear factor 1 B-type) translocation is widely reported, but alone is not sufficient for diagnosis [126,127,128]. In a small group of cases, *MYBL1* (MYB Proto-Oncogene Like 1) rearrangements occur (Table 1) [129,130].

The incidence ratio was reported to be 0.23 per one million person-years. The median age at diagnosis is the seventh decade, with a range of 14–94 years [5,123]. Slight female predominance is observed. The largest series of cases subsumed into a publication is 152 patients [5].

The lesion commonly presents a variably symptomatic, slow-growing, firm, skin-colored nodule [5,123]. Incidence rate per million population per 1,000,000 person-years was highest for tumors occurring on the face/head/neck (0.16), followed by trunk (0.04) and extremities (0.02). Among lesions occurring on the head and neck, most of them were found on the scalp and neck (33.6%), followed by external ear (29.8%), face (23%), eyelid (8.6%), and lip (4.8%) [5]. The tumor size typically ranges from 1 to 5 cm [123].

Histologically, ACC is composed of small basaloid epithelial tumor cells with a small to moderate amount of cytoplasm. The nuclei are rarely pleomorphic and have small or inconspicuous nucleoli. Cells exhibit either luminal epithelial differentiation or myoepithelial differentiation, with myoepithelial differentiation predominating [122]. Mitotic index is low, although cases with high-grade tumors may show >10 mitotic figures/mm^2^ [123]. The tumor is characterized by cribriform and less often tubular or solid growth patterns. The luminal areas, usually consisting of alternating eosinophilic or basophilic secretions, are present (Figure 6A1,A2). Like those of the salivary gland and elsewhere, cutaneous ACC shares a propensity for the perineural invasion, which was present in 76% of cases with local recurrence [123]. Differential diagnosis includes adenoid basal cell carcinoma; therefore, positive CD117, CK7, CEA, EMA, and CAM5.2 immunohistochemical staining is potentially useful in this distinction [131,132]. Neoplasm must also be differentiated from a metastatic ACC of other origin and other apocrine or eccrine neoplasms, including mucinous carcinoma or cribriform carcinoma [125]. CK5/6, CK15, and p63 have also been found to be useful distinguishing markers between primary cutaneous adenocarcinomas and cutaneous metastatic adenocarcinomas [127]. Other immunohistochemical markers used for differentiation from cutaneous secretory carcinoma include S100, mammaglobin, STAT5A, and NTRK3 (neurotrophic-3 growth factor receptor) [123,133].

Treatment generally includes surgical resection or MMS with a toluidine blue stain (instead of the usual hematoxylin-eosin stain), which some authors recommend. Higher sensitivity to identify perineural invasion may be beneficial for treatment outcomes [134]. Xu et al. reported that in a seven patients who underwent MMS, no local recurrence in a follow-up of 10–28 months occurred [125]. Some patients also receive adjuvant or therapeutic radiation therapy and chemotherapy (Table 2) [5,135]. Patients with tumors extending to the central skull base may benefit from proton beam radiotherapy. Pommier et al. reported 23 patients with ACC involving the skull base treated with proton beam radiation, where median dose of 75.9 Gy resulted in 93% local control of disease, 62% distant metastasis-free survival, and 77% overall survival in the median follow-up of 64 months [136]. One hundred and twenty patients with previously untreated ACC benefited from a combination of surgery and adjuvant radiotherapy (10-year overall survival of 57% versus 37% in patients with radiotherapy alone) in a study of Balamucki et al. [137]. Similar outcomes of a study of Douglas et al. on 151 patients treated for previously unirradiated nonmetastatic locally advanced and/or recurrent ACC showed that local-regional control was 43% after neutron radiotherapy alone, and 71% after subtotal resection and neutron radiotherapy [138]. Ikegawa et al. reported a case of a patient with a lesion on the frontal region, which was surgically removed and recurred three times: 23 years after initial diagnosis, 13 metastatic coin lesions in her lungs were found, and 2 cycles of chemotherapy consisted of cisplatin and adriamycin were administered. Complete remission was obtained; however, five months later, disease recurred, and the same treatment application was unsuccessful [139]. In a patient with ACC with lung metastases reported by Chang et al., 3 courses of palliative chemotherapy with cyclophosphamide, doxorubicin, and prednisolone were administered, which resulted in considerable decrease of the tumors, but the follow-up is unknown [140]. Other chemotherapy regimens were also successfully administered, including treatment consisted of cisplatin and 5-fluorouracil, or cisplatin and vinorelbine [141,142]. Lesions tend to recur locally in up to 50% of patients, but the metastatic potential is relatively low [123]. Distant metastases to the lung, bone, and soft tissue tend to be more common than regional lymph node spread [123]. Lesions may recur up to 35 years after primary excision and thus careful follow-up is recommended [143].

#### 2.1.11. Apocrine Carcinoma

Apocrine carcinoma (AC), also known as apocrine adenocarcinoma, is a rare malignancy deriving from sweat glands. Usually, it appears in regions with high apocrine glands’ density, such as the axilla, the anogenital area with nipples and head, including eyelids (from the glands of Moll), and the external auditory canal [144,145]. Few non-specific mutations have already been found (Table 1). Lesion may be associated with pre-existing naevus sebaceous [146,147,148].

The prevalence is unknown due to limited data. The largest series of affected patients in one paper includes 186 cases [144]. The disease presents in older patients, mainly in the seventh decade, but even patients affected in the second decade were observed. There is no racial or gender predilection.

The lesions may present as solid or cystic masses or subcutaneous nodules, usually with red and purple discoloration of the skin. The tumor surface might be ulcerative. The lesion is often locally advanced at the moment of diagnosis and infiltrates surrounding tissues [149]. The neoplasm may metastasize to regional lymph nodes and other organs, including lungs, liver, bone, and brain [144]. Although AC usually arises de novo, they can also originate from a pre-existing benign lesion, as in the case of adenocarcinoma derived from an apocrine cyst [150].

Histologically, the neoplastic cells are cuboidal to angulated in appearance with prominent nuclei and eosinophilic cytoplasm (Figure 6C1). They seem to form ill-defined tubular or glandular structures, partially resembling apocrine glands [145,149]. Cytologic atypia and mitosis may be presented [151]. Immunohistochemically, tumor cells reacted strongly to cytokeratin AE1/AE3, CK7, and CAM5.2 [145,149,150]. CK5/6 staining is also commonly positive (Figure 6C2). They are negative for cytokeratin 20 [152]. GCDFP-15 is a marker of apocrine differentiation and might distinguish between carcinoma of apocrine or eccrine origin [149,150]. The primary cutaneous apocrine carcinoma more frequently expresses EGFR, podoplanin, and p63 than other metastatic carcinomas, especially from the breast [153]. Primary cutaneous AC is usually adipophilin-negative, ER^+^, PR^+/−^, and HER2^−^, whereas mammary AC tends to be adipophilin-positive, ER^−^, PR^−^, and HER2^+^, which may be helpful in distinguishing cutaneous lesions from metastatic spread to the skin [153].

The therapeutic approach to apocrine adenocarcinoma includes surgery, radiation, and chemotherapy. Surgical treatment is usually performed at first, with wide margins, though the margin’s extent is not clarified (Table 2) [144,145]. Radiotherapy is claimed to be an additional treatment in patients with extensive tumors or for palliation of both bone and brain metastases, whereas chemotherapy, typically combinations of anthracyclines, taxanes, and platinum drugs, has been reported to be successful in cases with lymph node metastases [151]. Chemotherapy similar to the chemotherapeutic regimen for breast cancer has been successfully administered, for example, epirubicin and cyclophosphamide followed by oral fluorinatedpyrimidine in cases with metastatic AC of the axilla [154]. Morabito et al. reported a case of recurrent disease previously treated with surgery, radiotherapy, and chemotherapy (Al-Saraff schedule). The patient responded to a second-line systemic chemotherapy regimen consisting of methotrexate and bleomycin [155]. HER2 inhibitors (tanstuzumab) are also used [156,157]. There is one case in which the mTOR inhibitor was used (everolimus) after progression and confirmation that PIK3CA and KRAS mutations are present [158]. Biopsy of sentinel lymph node orlymphadenectomy could identify the clinically occult disease and may provide a better-defined prognosis, and thus should be recommended as the standard approach. Lymph node metastases are identified in 16% of patients with apocrine carcinoma. In a population of 186 patients reported by Hollowell et al., most patients presented with localized disease (57%), followed by locally advanced or regional (30%) and distant (5%) disease. In a review of 17 cases by Brown et al., common sites of disease spread were distant lymph nodes (axillary, subclavicular), skin, bone, brain, and the lungs. Survival time from the metastatic diagnosis ranged from approximately 1 to 4 years, with an average of 2.25 years [159]. The median overall survival is reported as 51.5 months, and localized disease has a good prognosis [144].

#### 2.1.12. Squamoid Eccrine Ductal Carcinoma

Squamoid eccrine ductal carcinoma (SEDC), also known as adenosquamous carcinoma of the skin, is a poorly documented neoplasm with ductal and squamous differentiation [14]. Ultraviolet radiation and immunosuppression can play a role in pathogenesis [4]. In a few cases, the development of SEDC was observed in patients with chronic lymphocytic leukemia [14].

The median age of patients with SEDC is 79.5 years old (range, 10 to 96), with a male predominance. White individuals have a higher risk than black. The most significant analysis is 30 patients subsumed into a publication [160].

SEDC is most frequently seen on the head and neck (~77%) as a solitary nodule or pustule (median size, 1.0 cm; range, 0.5 to 2.5 cm) [161]. As it was mentioned, neoplasm consists of ductal and squamous differentiation. Positive staining with EMA and CEA is typical of ductal tissue, and it helps to distinguish the SEDC from squamous cell carcinoma [14]. In one of the analyses, the authors listed the features along with the frequency of their occurrence in patients with SEDC. In the macroscopic and microscopic evaluation, the cytologic atypia was moderate to severe, ulceration (47%), necrosis (23%), and perineural and lymphovascular infiltration (27% and 6%, respectively). Distant metastasis is relatively rare [160].

Treatment recommendations include WLE or MMS (which may be beneficial, especially in view of the high incidence of facial tumors), clinical screening, and careful follow-up (Table 2) [162]. The local recurrence rate is 25%, not related to completeness of resection after median 29-month follow-up. In a recent case series, the metastatic spread was observed in 13% of cases, and one patient died of metastatic disease [160]. Saraiva et al. reported the patient who underwent adjuvant radiotherapy (in linear accelerator with an energy beam of 6 mV at a dose of 66 Gy in two cycles) because of double incomplete excision. Five months later, recurrence was observed, and the complete surgical excision was performed [161]. No chemotherapeutic has been assessed as the treatment.

#### 2.1.13. Syringocystadenocarcinoma Papilliferum

Syringocystadenocarcinoma papilliferum (SCACP) is the malignant counterpart of syringocystadenoma papilliferum (SCAP). Usually, it develops on the scalp in a long-standing lesion identified clinically as SCAP, although due to the rarity of this tumor, etiology and origin are not clarified [163,164]. Lesions are found in the same locations as SCAP. SCACP is thought to be a low-grade malignancy with a favorable prognosis. Locoregional lymphatic metastases are present in about 20% of patients, and distant metastases were reported in only a few cases [165].

SCACP usually affects middle-aged or elderly individuals and does not seem to have a gender predilection. To date, only about 50 cases of SCACP have been described in the literature [164]. The most significant series of cases is ten patients subsumed into a publication [165].

SCACP presents typically as an asymptomatic long-standing flesh-colored to a hyperpigmented lesion, flat, nodular, or cystic, and sometimes with the ulcerated surface [164]. Its size varies from 0.5 to 13 cm. The tumor may also be associated with cystic degeneration, discharge, or pain. The majority of reported SCACP presented as adenocarcinoma in situ and invasive adenocarcinoma, but sometimes it could also exhibit histological variabilities, such as invasive squamous cell carcinoma or pagetoid spread [165,166].

Histologically, syringocystadenocarcinoma papilliferum has many structural similarities with SCAP. In the upper portion, numerous deep invaginations are containing papillary structures lined by a two-layered epithelium. The luminal layer is composed of columnar cells with oval nuclei and abundant eosinophilic cytoplasm, whereas the outer layer consists of small cuboidal cells with oval nuclei and scanty cytoplasm. Stroma is densely infiltrated by plasma cells and lymphoid cells. In the deeper parts of the tumor, numerous irregular neoplastic tubular and cystic structures, lined by monolayered or multilayered atypical cells with enlarged and hyperchromatic nuclei, diffusely infiltrate the whole dermis in an invasive growth pattern. Occasionally, numerous normal apocrine glands can be found [163]. The proliferative index is high. Basal/myoepithelial cells of the outer layer might be highlighted by p63 staining. GCDFP-15 may be expressed in the in situ areas and components of invasive adenocarcinoma. Other immunohistochemical markers for epithelial cells include EMA, CK7, and CAM5.2 [164,165,167]. In the limited number of cases, an extramammary Paget disease should be excluded since lesions suggesting SCACP in situ may show pagetoid spread [168].

The therapeutic approach to SCACP is based on a WLE. MMS may be performed as well [169]. In some cases, radiation (to the local recurrence after re-excision) or chemoradiation (cisplatin with irradiation up to a dose of 59.4 Gy in 33 fractions) was successfully performed (Table 2) [170,171]. However, recurrences are possible, and until now, there are no reliable clinical or histological indicators to determine the prognosis. Locoregional lymphatic spread rate is estimated to be 18.4%, and single metastatic disease cases have been reported to date [165,172].

#### 2.1.14. Secretory Carcinoma

Secretory carcinoma (SCA), also known as primary cutaneous mammary analog secretory carcinoma, is a low-grade malignant neoplasm. Since the primary description of SCA in the breast, neoplasms with similar features have been reported in multiple non-mammary sites, including the salivary glands, lacrimal gland, sinuses, lip, buccal mucosa thyroid gland, lung, and skin [173,174]. Cutaneous SCA harbors the *ETV6-NTRK3* (ETS Variant Transcription Factor 6) fusion gene, widely reported in the tumor of salivary glands and breast [175,176,177]. However, *ETV6-NTRK3* rearrangement is not unique to SCA because it is also found in other neoplasms, such as congenital fibrosarcoma, cellular or mixed mesoblastic nephroma, myeloid leukemia, or well-differentiated papillary thyroid carcinoma [178]. A case with *NFIX-PKN1* (nuclear factor 1 X type-serine/threonine-protein kinase N1) translocation and the other with heterozygous deletion of ETV6 in 25% of cells has also been reported (Table 1) [179,180].

The first case was described by Brandt et al. in 2009, and since then, less than 30 cases have been reported [181]. The tumor usually occurs in adults (mean: 51.8), although lesions in younger patients have been reported, and it occurs mainly in females [179,182]. The main location is the axilla, followed by the neck and lip.

Grossly, MASC presents a solitary, well-circumscribed, but unencapsulated tumor with a variable cystic component. Cutaneous SCA frequently involves the axilla, followed by head and neck, flank, and forearm [183]. The size usually ranges from 0.4 to 6 cm [183].

Histologically, the lesion shows variable growth patterns, including solid, cystic, tubular, and (pseudo)papillary. SCA is characterized by the formation of round to ovoid microcysts full of pink fluid that gives a homogeneous positive periodic acid-Schiff (PAS) reaction. Neoplastic cells contain pale and ovoid nuclei with eosinophilic and/or vacuolated cytoplasm. The perineural invasion may be seen, but significant cytologic atypia, tumor necrosis, or numerous mitotic figures are absent. Immunohistochemical staining shows positivity for keratins (mainly CK7), S100 protein, vimentin, mammaglobin, GATA-3 (GATA Binding Protein 3), NTRK, and STAT5A. Cells are frequently negative for basal/myoepithelial markers, with variable expression of calponin and p63 [176]. Positive staining with EGFR, EMA, MUC1 (mucin 1), MUC4 (mucin 4), SOX10 (SRY-Box Transcription Factor 10), and GCDFP-15, among others, have also been reported, although their expression is variable and non-specific [175,183].

The therapeutic approach includes surgical excision. The sentinel lymph node mapping is not indicated in primary cutaneous secretory carcinoma [184]. No recurrent or metastatic disease cases have been reported yet, and the prognosis is good after complete excision (Table 2) [183].

#### 2.1.15. Cribriform Carcinoma

Cutaneous cribriform carcinoma, first described by Requena et al. in 1998, is a low-grade malignancy with apocrine differentiation [185]. However, the lesion does not tend to occur in areas with a high density of apocrine glands, including the axilla and anogenital region. Typical sites are extremities with infrequent distribution on the head and neck [186]. A solid variant of this carcinoma has been distinguished [187,188]. No specific mutations have been reported.

The average age of patients is the fifth decade (mean = 47 years old), and the lesion occurs in adults of all ages. There is a female predilection (F:M = 2:1) [186,189]. The biggest series of cases is 26 patients subsumed into a publication [190].

The lesion typically presents as a symmetric, well-circumscribed, skin-colored nodule, ranging in size from 1 up to 3 cm [190]. Histologically, the tumor consists of cribriform spaces and solid areas formed by multiple interconnected nests of neoplastic cells presenting with hyperchromatic round or oval nuclei, and granular chromatin with scant eosinophilic cytoplasm. The cells lining the luminal spaces range from cuboidal to markedly flattened, with elongated nuclei. No appreciable myoepithelial layer is reported. Small intraluminal micropapillae might also be seen. Structures are set in desmoplastic stroma composed of collagen bundles. In a minority of lumina, scattered eosinophilic secretions may be observed. Decapitation secretion might be focally present. Lymphoid aggregates are widely observed [191]. The lesion may present with a pushing border into the subcutaneous fat. Infrequent mitoses with occasional necrosis occur. Lymphovascular or perineural invasion is not reported. Immunohistochemically, the tumor is usually positive for AE1/AE3, CAM5.2, CK5/6, CK7, CD117, MNF116, and BerEp4, and variably positive for EMA, CEA, p53, S100 protein, and estrogen receptor [192,193,194]. Stains such as GCDFP-15, ER, PR, CK20, CDX-2 (caudal type homeobox 2 protein), PSA (prostate-specific antigen), and PAX-8 (paired box gene 8) may help support metastasis diagnosis to distinguish them from primary cutaneous lesions; forasmuch, lesions with the cribriform pattern are seen in other organs [190,195]. Differential diagnosis also includes tubular adenoma, which can be distinguished by a myoepithelial layer around the tumor nests [189]. GATA3 expression has not been detected, as opposed to breast cancer metastasis [186].

The first-line therapeutic approach is surgical resection. Reported margins reach up to 5 mm. In some cases, MMS is performed to reduce the risk of recurrence. Sentinel lymph node biopsy is sometimes performed, although it is usually negative. There has been no metastases report with singular cases of recurrent disease treated by reoperation because of incomplete primary resection (Table 2). Due to the low number of reported cases and uncertain ability to form metastases, long-term follow-up is recommended [189,193,195].

#### 2.1.16. Signet-Ring Cell/Histiocytoid Carcinoma

Signet-ring cell/histiocytoid carcinoma (SRCHC) is an extremely rare adnexal neoplasm. It is considered equivalent to the histiocytoid variant of invasive lobular carcinoma of the breast. Mutation of PIK3CA, NTRK3, CDKN1B, HER2/neu, and CDH1, the genes encoding E-cadherin 1, have been reported, and they are known to be pathogenic in invasive lobular carcinoma of the breast (Table 1) [23,24,196]. The targeted therapy may help treat locally aggressive disease, but further studies are needed to assess this approach.

The lesion shows a male predilection, and it usually appears in adults in their seventh decade (mean 67.1 years old) [197]. About 50 cases have been reported in the English literature [23].

The lesion usually presents as a slow-growing, firm, solitary cutaneous or subcutaneous nodule, accompanied by painless infiltration with thickening and eyelid swelling. Sometimes, infiltration may spread circumferentially around the eyelid, leading to a “monocle-like” appearance. The second most frequent location is the axilla, with more than 10 cases reported. The axillary lesion is considered less aggressive than its palpebral counterpart [23]. Histologically, it is characterized by diffuse proliferation of single or cords of histiocytoid carcinoma cells and the presence of signet-ring cells in the dermis and/or subcutis, usually without epidermal involvement. The histiocytoid cells can be misinterpreted as macrophages, especially with the presence of other inflammatory cells. MUC6 positivity on immunohistochemical staining may suggest histogenesis from the glands of Moll. Other immunohistochemical features are epithelial and apocrine markers, including cytokeratins, CEA, EMA, GCDFP-15, human milk fat globule, Ber-EP4, GATA-3, MUC-1, with variable CD138, α-SMA (alpha-smooth muscle actin), CK20, tissue-specific transcription factor 1, MUC-2, podoplanin, N-cadherin, Her-2, ER, and PR expression [198,199,200]. E-cadherin is immuno-positive in most cases of primary cutaneous SRCHC, in contrast to histiocytoid breast carcinomas. Expression of AR is considered consistent with the apocrine nature of SRCHC [23]. However, lack of morphological apocrine secretion and GCDFP-15 immunoreactivity reported in normal eccrine glands may indicate another origin [201]. Differential diagnosis includes metastatic adenocarcinoma, arising from breast, lung, or gastrointestinal tract, and there are no definitive markers to distinguish between primary and metastatic disease, so it is important to rule out another primary tumor before the diagnosis can be established [197].

The recommended therapeutic approach is WLE, sometimes with exenteration of the orbit. The tumor tends to recur or metastasize (~30% over 10-year follow-up); therefore, adjuvant radiotherapy (50–64 Gy) can be combined with chemotherapy (5-fluorouracil, doxorubicin, cyclophosphamide, methotrexate, melphalan, and 13-cis-retinoic acid without significant impact on survival), anti-estrogen (tamoxifen), or anti-androgen therapy, depending on the tumor markers expressed, which were applied in some cases with variable outcomes (Table 2) [24,197,198,199]. Regional lymph node metastases are most common, but they can occur on the skin of the head, neck, trunk, respiratory tract, vulva, bone marrow, spine, and parotid gland. Furthermore, most relapses have been associated with incomplete tumor excision or orbital involvement [23,199]. Involvement of contralateral eyelid is also possible [202,203]. Palakkamanil et al. administered adjuvant radiotherapy (50.4 Gy in 28 fractions) because of positive surgical margin 3 months after operation and reconstruction of the eyelid without evidence of recurrence after 4 years of follow-up [204].

### 2.2. Benign Tumors with Apocrine or Eccrine Differentiation

#### 2.2.1. Hidrocystoma/Cystadenoma

Hidrocystoma/cystadenoma is a benign lesion mainly arising from the duct of the sweat gland. It can have a simple cystic (hidrocystoma) or more complex (cystadenoma) architecture [205]. The etiology is unknown; however, eyelid apocrine hidrocystomas may be encountered in Schopf-Schulz-Passarge syndrome (Table 1) [16,17].

Apocrine hidrocystoma is mainly observed in adults aged 30 to 70 years. There is no correlation between incidence and sex, race, or geographical region [206].

**Figure 7:**
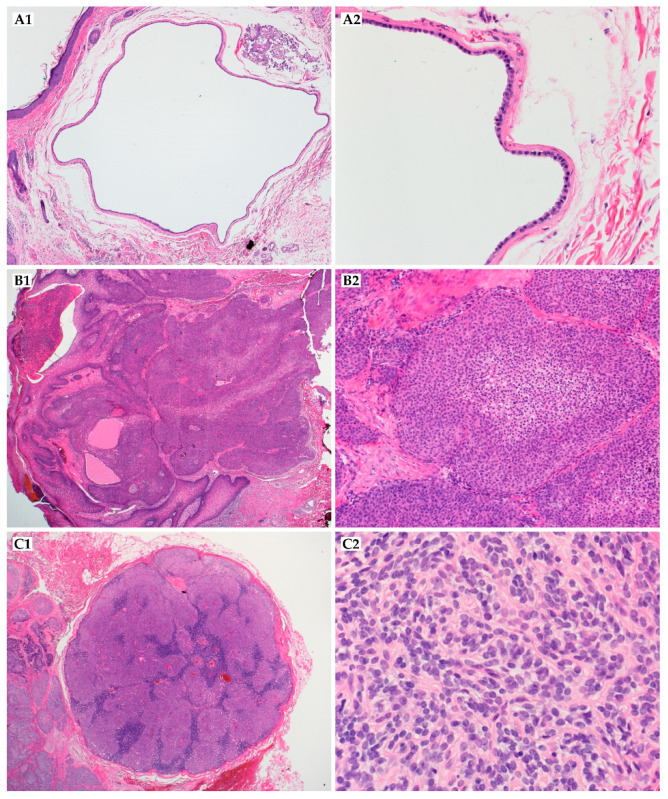
Benign tumors with apocrine or eccrine differentiation I. (**A1**) (20×, HE) and (**A2**) (200×, HE): Hidrocystoma: tumor composed of cystic spaces lined with cuboidal epithelium. (**B1**) (40×, HE) and (**B2**) (100×, HE): Poroma, a well-circumscribed lesion with poroid and cuticular cells. (**C1**) (40×, HE) and (**C2**) (400×, HE): Spiradenoma, well-circumscribed nodules, which consist of basaloid cells with the visible basement membrane material.

The size of skin lesions ranges from 3 to 15 mm in diameter [207]. On the microscopic level, apocrine hidrocystomas are well-circumscribed cystic lesions and enclosed by fibrotic tissue (Figure 7A1,A2). They have a low mitotic index with little to no nuclear atypia [208]. In immunohistochemical staining, the luminal cells in hidrocystomas or cystadenomas are positive for EMA and CEA, and the myoepithelial elements are positive for S100 protein [206,209]. Other markers found in these lesions include GCDFP15, as well as various keratins [210,211,212].

The most common treatment of hidrocystoma is complete surgical excision with narrow margins because of the lesion’s benign nature (Table 2). The hypertonic glucose sclerotherapy, which followed the cyst puncture, was used successfully to treat eyelid apocrine hidrocystoma. Trichloroacetic acid injection (followed by aspiration) after cyst puncture may also be an alternative to surgery. Other therapeutic methods that may be used are electrodesiccation, radiofrequency ablation, and carbon dioxide laser treatment [206,213]. The topical use of botulinum peptide is useful in some cases [214].

#### 2.2.2. Syringoma

Syringoma is a benign, usually eccrine sweat gland neoplasm. Contrary to what was believed for a long time, the origin is not the intraepidermal section of the sweat duct but the intraglandular duct [215]. Although the most common localized involvement site is the periorbital area, other locations, including the genital region, trunk, or scalp, are also widely reported [29,216]. No specific mutations have been found. However, syringoma has been found in association with several systemic syndromes, especially Down syndrome or diabetes mellitus, Marfan syndrome, Ehler-Danlos syndrome, Nicolau-Balus syndrome, sarcoidosis, cicatricial alopecia, and alopecia areata [29,30]. Other cases were associated with anti-epileptic medications [217,218] or hyperthyroidism [219,220]. Some researchers suggest that syringoma may start as a primary inflammatory eccrine reaction, though it has not been proven [221,222]. Friedman and Butler distinguished four clinical variants of syringoma based on clinical features: a localized form, a form associated with Down syndrome, a familial form, and a generalized variant, including multiple and eruptive syringomas [29].

The prevalence is higher for women, though the lack of female predominance in familial cases and immunohistochemical studies of PR and ER have not been consistent in confirming hormonal involvement [29]. It is believed that the eruptive variant is most common in young women [223]. More than 800 cases of syringoma have been reported, and the largest series of affected patients in one paper includes 244 cases [29,216].

The lesion appears as a small, firm, asymptomatic papule, typically flesh-colored or yellowish. The average size is 1–3 mm. Usually, the tumor presents with a singular lesion; however, the eruptive variant consists of multiple papules. The differential diagnosis includes milia, xanthoma, trichoepithelioma, xanthelasma and hidrocystoma, cutaneous mastocytosis, fibrofolliculomas, vellus hair cysts, angiofibromas, or fibroelasticpapulosis [29].

Histological examination shows nests of eccrine ducts, tadpole-like structures embedded in the fibrous stroma, and clear cell changes of epithelial cells. Acanthosis, hyperpigmentation of basal keratinocytes, keratin cyst/milia-like structures, and telangiectasia of superficial vessels may also be present [224]. The ducts sometimes presents a characteristic tadpoil tail pattern. Although calcium deposition is a unique feature of syringoma in general, it is seen in patients with Down syndrome, and because of the risk of progression to calcinosis cutis, it necessitates prompt treatment [29].

Lesions are considered benign and nonprogressive, so the purpose of treatment is to improve cosmetic appearance. The therapeutic approach includes surgical interventions and other medical therapies, such as CO_2_ laser or retinoids (Table 2) [76,225,226]. Nevertheless, no single treatment has proven to be consistently efficacious. Moreover, recurrence, scarring, and dyspigmentation are common complications of these interventions [29].

#### 2.2.3. Poroma

A poroma is a benign adnexal neoplasm that originates from the intraepidermal portion of the sweat gland duct, called acrosyringium. Initially, it was described as a tumor arising from the eccrine sweat gland; however, the apocrine components may be found. It may develop into porocarcinoma through degenerative progression, and the transformation rate is about 18%. In a small percentage of poromas, *HRAS* mutations have been reported. Highly recurrent *YAP1-MAML2* and *YAP1-NUTM1* fusions in most of the poromas were found (Table 1) [28].

Poroma constitutes approximately 10% of sweat gland tumors. It tends to occur in the middle age to the elderly population and does not appear to have a predilection for race or sex. Long-term radiation exposure, including electron beam therapy, and other skin lesions, such as hypohidrosis ectodermal dysplasia, Bowen’s disease, or naevus sebaceous, may be a risk factor for the development of poroma [11].

Poroma can be located on almost any cutaneous surface with a predilection to acral sites. Intradermal neoplasm commonly involves the head and neck region. Poroma presents with papule, plaque, or nodule, and it is white to even blue. Its surface may be ulcerative. Generally, it is slow-growing and asymptomatic for the patient, although some patients might experience itching or pain. Sometimes poromatosis is present, with multiple, widespread lesions at the moment of diagnosis [11,227]. In the dermatoscopic examination, a polymorphic vascular pattern is probably the most prominent finding facilitative in diagnosis. However, it may be seen in other lesions, including melanoma, so further investigation is needed. Nonetheless, the leaf- and the flower-like patterns seem relatively unique for poroma [11].

In histological examination, poroma is composed of cuticular and poroid cells that commonly extend from the basal epidermis into the dermal layer (Figure 7B1,B2). The cells may form ducts or tubules. Even though it is benign in nature, a variable number of mitotic figures, highly vascularized stroma, and necrosis may be found, mainly in superficially traumatized lesions [11]. The presence of both eccrine and apocrine components can be identified by CEA immunostaining. EMA also highlights ductal differentiation. Contrary to eccrine poroma, an apocrine one may be presented with homogeneous eosinophilic intraluminal secretion and lining cells with eosinophilic cytoplasm. Distinctive features comprise the presence of sebaceous cells and peripheral areas with follicular differentiation.

As a benign lesion, poroma is curable by simple excision, and recurrence is rare (Table 2) [11].

#### 2.2.4. Syringofibroadenoma

Syringofibroadenoma, also called acrosyringial naevus, or eccrine syringofibroadenomatous hyperplasia, is considered as a benign eccrine neoplasm. The origin is the acrosyringeal part of the eccrine duct; however, this entity’s histogenesis remains controversial due to cytokeratin expression, suggesting eccrine duct origin [228,229]. Often, it is encountered in association with other dermatoses or skin tumors [230,231,232]. Multiple tumors of the palms and soles are described as distinctive features of Schöpf-Schulz-Passarge syndrome [233]. HPV-10 might also play a role in developing eccrine syringofibroadenomas [31]. Despite being considered a benign condition, either a hamartoma or reactive hyperplasia, some evidence indicates that long-standing syringofibroadenoma may undergo metaplasia [230,234].

Less than 100 cases have been reported [228]. The age of onset varies, and the lesion typically presents between 16 and 80 years, although solitary lesions are seen most commonly in the seventh and eighth decades [235]. Sex predilection is unknown due to limited data.

Neoplasm is characterized by polymorphous clinical presentation ranging from a solitary papule or nodule to multiple lesions with a linear arrangement, known as eccrine syringofibroadenomatosis. The lesion is usually slow-growing and flesh- to reddish-colored [236]. The tumor is typically found on the lower extremities, sometimes with nail involvement [229]. Histologically, a well-circumscribed tumor is composed of proliferating, anastomosing cords of monomorphous epithelial cells surrounded by abundant fibrous tissue. The lesion may show staining for CKAE1/AE3, CK5/6, CEA, EMA, CAM 5.2, CK19, and others [228,237,238,239].

The therapeutic approach depends on the number, location, and resectability of the lesions (Table 2). The solitary tumor is surgically excised. In unresectable cases, monitoring and generous sampling are recommended to exclude malignant transformation [236,240]. Spontaneous regression of reactive subtype following successful treatment of the underlying inflammatory condition has been reported. That subtype may therefore be regarded as secondary, non-autonomous epidermal hyperplasia [240]. Other therapeutic options include cryotherapy and CO_2_ laser therapy, photodynamic therapy, curettage, electrodesiccation, and radiotherapy, as well as topical and systemic retinoid acids, 5-fluorouracil and imiquimod, with variable patient outcomes [228,231,235,236,241,242].

#### 2.2.5. Hidradenoma

Hidradenoma is a benign tumor, presenting as a slow-growing nodular mass, consisting of apocrine and eccrine components [243]. In approximately 50% of cases, the presence of t(11;19) translocation with an associated novel fusion oncogene (*CRTC1-MAML2*) was reported. The *CRTC1-MAML2* oncogene acts as a transcription factor on Notch and CREB (cAMP response element-binding protein) regulatory pathways, disrupting normal cell cycle and differentiation, contributing to cancerogenesis (Table 1) [244].

There is a slight female predominance, and the mean age at diagnosis is approximately 37 years old, but it can occur at any age [245].

Hidradenoma is an asymptomatic, solitary nodule, usually less than 2–3 cm in diameter. The differential diagnosis of hidradenoma should be taken into account for the other clear cell neoplasms. Hidradenoma is stained positive for several keratins, such as CK7 and CK5/6, p63, EMA, and CEA, the latter underlining ductular structures inside the tumor [243,246].

An optimal treatment protocol has not been established. In most cases, the recommended form of treatment is total excision (Table 2) [247].

#### 2.2.6. Spiradenoma

Spiradenomas are well-differentiated, benign, and dermal neoplasms that arise from the hair follicle bulge rather than the eccrine sweat gland, based on an immunohistochemical study of stem cell markers and with CD200 [248]. The pathogenesis is not entirely understood, but a defect in the tumor suppressor gene, *CYLD*, is thought to contribute to their development in Brooke-Spiegler syndrome, which also features multiple spiradenomas [249]. Additionally, derangements in intercellular bridge proteins that maintain epithelial organization, including claudin-4, cadherin, and beta-catenin, have also been suggested as contributing to neoplasm formation (Table 1) [250].

The spiradenomas are usually benign and most often occur in patients between the ages of 15 and 35, though cases have been reported at any age. The risk of malignant transformation increases with age. Spiradenomas usually arise on the head, neck, and trunk; however, cases in other areas such as the breast have occurred. The exact incidence of benign tumors is not known. The link between race, sex, and incidence does not exist. The risk of malignant transformation increases with age (significantly above 50 years old) [251].

The spiradenomas typically present as dermal or subcutaneous nodules on any area, though more commonly are seen on the head, neck, and trunk, and less commonly on the arms and legs. On physical examination, lesions are typically small, though they can grow to several centimeters in diameter and have a blue, gray, or purple hue. The spiradenomas may be painful, but it is not necessarily a defining characteristic [252]. Although the typical presentation is a solitary lesion, there can be linear or grouped spiradenomas. Spiradenomas are well-circumscribed and built from nodules of two types of basaloid cells (light cells in the center of nodules and dark cells in the peripheral parts). Basement membrane material usually separates the islets of tumor cells (Figure 7C1,C2).

Conservative surgical excision of spiradenomas is therefore recommended (Table 2). In cases of multiple spiradenomas, adjunctive treatment with CO_2_ laser may be employed after surgical debulking.

#### 2.2.7. Cylindroma

Cutaneous cylindroma is a rare benign adnexal neoplasm with a microscopic mosaic pattern. In sporadic cases, the expression of fusion transcripts, *MYB-NFIB,* and mutation in the *CYLD* gene are observed [253,254]. The *MYB* is an oncogene that, when fused with *NFIB*, a transcription factor gene forms an oncoprotein that can encourage a neoplastic process [253]. The majority of familial cases are associated with mutations in the *CYLD* tumor suppressor gene (Table 1) [255]. The histogenesis of cylindroma is not entirely clarified.

Cylindromas occur in elderly patients, with female predominance (female to male ratio, 2:1). Familial cases are associated with Brooke-Spiegler syndrome (BSS) [256].

In clinical examination, cylindroma presents firm, nodular tumors, with a size diameter of about 2–6 mm, showing slow growth.

**Figure 8:**
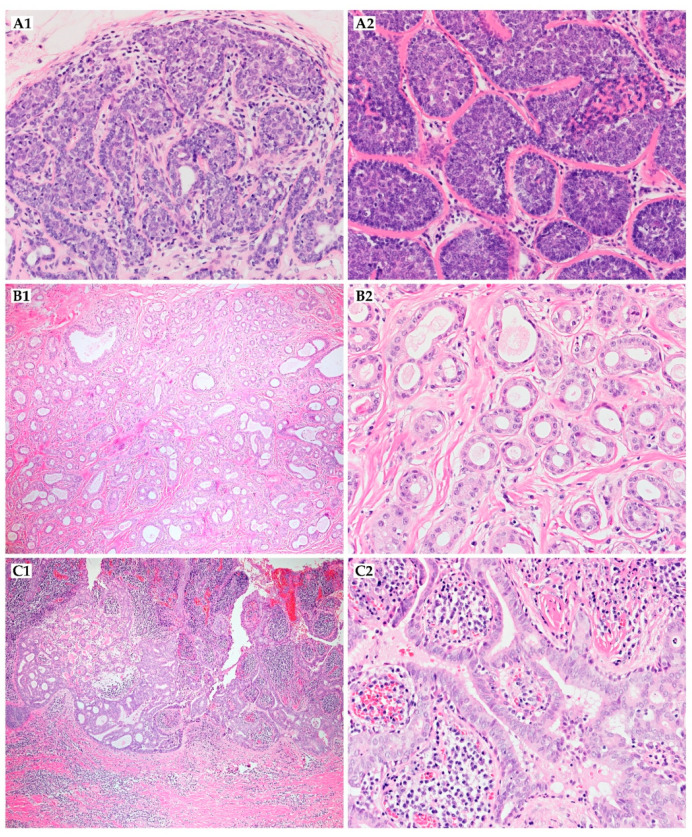
Benign tumors with apocrine or eccrine differentiation II. (**A1**) (100×, HE) and (**A2**) (200×, HE): Cylindroma, microscopic mosaic pattern with the jigsaw puzzle arrangement and surrounding of the prominent basement membrane. (**B1**) (20×, HE) and (**B2**) (200×, HE): Tubular adenoma composed of tubules and cystic structures. (**C1**) (20×, HE) and (**C2**) (200×, HE): Syringocystadenoma papilliferum consisting of two layers of apocrine cells which are forming papillary projections.

On a microscopic view, this neoplasm consists of small, irregularly shaped aggregations of basaloid keratinocytes closely opposed to one another, which are surrounded by a hyalinized basement membrane (Figure 8A1,A2) [256]. All cylindromas stain with PAS.

The complete excision is the preferred treatment (Table 2). Late local recurrence has been documented, so subsequent follow-up should be considered [257]. Large lesions should be imaged before planning treatment to determine vascularity and involvement of surrounding tissues, including underlying osseous structures. Some authors recommend topical aspirin to prevent recurrence after excision [256,258].

#### 2.2.8. Tubular Adenoma

Tubular adenoma (TA), also known as tubular papillary adenoma, papillary eccrine adenoma, or tubular apocrine adenoma, is a rare benign tumor of sweat glands. In most cases, the lesion shows an apocrine differentiation, but eccrine differentiation may be present as well [259]. The lesion is often associated with syringocystadenoma papilliferum or naevus sebaceous, and also morphological overlap between TA and SCAP has been reported [260,261,262]. BRAF and KRAS mutations may be present (Table 1) [263].

Less than 100 cases have been reported [259]. Neoplasm has a wide age distribution and a female predominance [264].

Lesions are most commonly seen in the extremities or the head and neck region, especially the scalp [263]. The tumor usually presents as an asymptomatic, well-defined, red to brown nodule, with size ranges from <1 to 7 cm [264].

Histologically, a well-circumscribed tumor is characterized by lobules composed of tubular glands in the dermis, sometimes with subcutaneous extension, with or without papillae. The lining of the glands consists of cuboidal luminal epithelial cells with eosinophilic cytoplasm and round or ovoid nuclei with small nucleoli (Figure 8B1,B2). Around the luminal cells, myoepithelial cells are present. Fibrous stroma is often sclerotic with a mild mononuclear cell infiltrating. Decapitation secretion is found in neoplasms arising from apocrine glands [263]. The neoplasm may be accompanied by follicular and sebaceous differentiation [261]. TA may arise in the deep dermis without any epidermal connection, or, in other cases, it can be more superficially located with or without a connection to the epidermis [260]. Dermatoscopic features include milia-like cysts, ulcerations, and arborizing, hairpin, glomerular vessels [264]. Immunohistochemical findings include CEA and EMA (luminal cells of tubules) and S100, p63, and calponin with SMA (outer cell layer) [189,262,265,266,267].

The lesion is considered benign, so the therapeutic approach includes surgical excision (Table 2) [264].

#### 2.2.9. Syringocystadenoma Papilliferum

Syringocystadenoma papilliferum (SCAP) is a benign adnexal neoplasm with apocrine differentiation, which is usually found on the head and neck skin at a young age. Other locations can be affected in the onset of puberty [268,269]. It may occur sporadically, but these lesions are commonly associated with other lesions and tumors, including naevus sebaceous [12,13], condyloma acuminatum [33], giant comedo [270], and verrucous cyst or carcinoma [271,272]. *BRAF V600E* mutation or activating mutations in *HRAS* (or less often *KRAS*) have been reported in sporadic forms. In contrast, types developing from naevus sebaceous carry the same *HRAS* mutation as the underlying naevus (Table 1) [273,274,275,276,277]. In sporadic cases and lesions arising in naevus sebaceous, the HPV DNA has been detected [32,33,34].

SCAP usually occurs at birth or a young age, and later mainly in the third or fourth decades, more commonly in females. The incidence is unknown. The largest series of affected patients in one paper includes 16 cases [34].

SCAP presents mostly with an asymptomatic plaque-like lesion that gradually becomes more elevated and assumes a verrucous or papillomatous configuration. Central ulceration or crusting may occur, and it becomes the main reason to misdiagnose the lesion as basal cell carcinoma [268]. Dermoscopically, polymorphous vessels were the prevalent feature [278].

Histologically, a uni- or multi-nodular lesion of SCAP is composed of branching cystic glandular structures beneath the epidermis, usually connected with dilated follicular infundibula (Figure 8C1,C2). The cystic cavities are filled with numerous protruding papillary structures lined by an inner layer of cuboidal epithelial cells, showing decapitation secretion features, and an outer one composed of columnar myoepithelial cells. The stroma of those papillary structures is highly vascularized and rich in plasma cells [271]. Mucinous metaplasia may be present. A morphological overlap between tubular adenoma and SCAP due to a lack of universally accepted diagnostic criteria has been reported [260]. The histopathological differential diagnosis also includes hidradenoma papilliferum, although it lacks epidermis connection, plasma cell infiltration, and is observed in the vulva region [279]. In immunohistochemical stains, the luminal columnar cells are mostly positive for CK7 and CK19, and they show heterogeneous expression of CK1/5/10/14, CK5/8, and CK14, and anti-CEA and anti-EMA antibodies highlight the apical surface of those cells in various degrees. The basal cells almost constantly express CK1/5/10/14, CK5/8, CK7, CK14, and vimentin; in some cases, CK19 and α-SMA may also be positive [280].

SCAP is unlikely to regress, and it frequently enlarges, so the evidence suggests that the lesion should be managed with complete conservative excision (Table 2) [281]. However, recurrence is possible [268]. CO_2_ laser excision of SCAP of the head and neck remains a clinical treatment option in anatomic areas unfavorable to excision and grafting [282].

#### 2.2.10. Cutaneous Mixed Tumor

Mixed tumors of the skin, also known as chondroid syringomas, are relatively common adnexal neoplasms. Usually, they derivate from apocrine glands, although tumors with eccrine origin are also reported [283]. In most cases, lesions are considered benign, however a malignant variant is also reported. Several mutations have been identified, including *PLAG1* (pleiomorphic adenoma gene 1 protein) or *EWSR1* (Ewing sarcoma breakpoint region 1) gene rearrangements, also found in myoepitheliomas, similar to pleomorphic adenomas (Table 1) [26,27].

Patients with mixed tumors are usually middle-aged, and the reported age range is 10–96 years. There is a male predilection. The cutaneous mixed tumor incidence is rare, comprising <0.01% of all primary skin tumors [80]. The most extensive series of affected patients in one paper includes 244 cases of apocrine tumor [284] and 50 cases of eccrine variants [283].

Typically, patients present with small solitary asymptomatic nodules that tend to involve the face, especially the nose, upper lip, and cheek. However, other locations, including trunk and extremities, are also reported. In most cases, the tumor is well-circumscribed, with or without a capsule/pseudo-capsule and a white homogeneous cut surface. Lesions are <3 cm in size, although a 10 cm lesion has been reported in the literature [80,284].

Histologically, mixed cutaneous tumors resemble mixed tumors of the salivary or other glands (pleomorphic adenoma). Most cases exhibit the classic appearance of interconnected, double-layered ducts embedded in chondromyxoid or fibromyxoid matrix. However, in some cases, tubular structures are intersected in reticulated or retiform patterns (“an appearance like a cluster of grapes or a bunch of berries formed by epithelial cells”). Apocrine mixed tumors often exhibit decapitation secretion, a feature of the apocrine epithelium. Moreover, follicular and/or sebaceous differentiation may also be present. In contrast, eccrine tumors show neither decapitation secretion nor follicular/sebaceous differentiation. Other changes in the epithelial component are also identified, such as squamous cell or clear cell metaplasia [284]. Epithelial cells are diffusely positive for CK7 and S100 protein, with constant negativity for p63 and CK5/6 [283]. Myoepithelial cells may transform into hyaline cells or other types [284]. Myoepithelial differentiation is frequently present and can be confirmed with myoepithelial markers, including smooth muscle actin or calponin [283]. Prominent myoepithelial differentiation may indicate myoepithelioma.

In most of the reported cases, lesions were surgically excised, though some of them needed re-excision after incomplete extirpation [283,284]. Despite considering them as benign lesions, they may regrow (Table 2) [80,285]. Excisional biopsy remains the most reliable method for preventing recurrence. Excision techniques that do not allow pathological evaluation of the entire specimen should be avoided [286].

#### 2.2.11. Myoepithelioma

Myoepitheliomas are considered benign soft tissue neoplasms consisting of myoepithelial cells. They are usually found in salivary glands and soft tissues, although primary cutaneous tumors are increasingly reported [287]. *EWSR1* rearrangements, also described in mixed tumors, occur in myoepitheliomas in a subset of cases [288,289]. Syncytial myoepithelioma is known to be a distinct cutaneous variant, which in most cases is characterized by recurrent *EWSR1-PBX3* (pre-B-cell leukemia transcription factor 3) fusion (Table 1) [290,291].

Myoepithelial carcinoma is an exceedingly rare cutaneous neoplasm. It can occur de novo or arise within a pre-existing myoepithelioma [292].

Myoepithelioma most commonly arises in adult patients, although a wide age range is observed [290]. There is a male preponderance [293]. Only a few series have been reported, including 38 patients as the biggest group of cases subsumed into a publication [286,287,289,293,294]. Less than 20 cases of myoepithelial carcinoma have been reported [294,295].

Lesions may occur in various parts of the body, with a predilection for extremities [287]. Tumor usually presents as a painless nodule, ranging in size from 0.3 to 18.0 cm [287,288,290,294]. Myoepithelial carcinoma ranges in size from 0.7 to 7.0 cm, and the lesions are usually tender and ulcerated, sometimes accompanied by satellite nodules [294].

Histologically, the tumor is composed exclusively of myoepithelial cells, resembling the myoepithelial component of a mixed tumor [287]. Myoepithelial cells may show spindled, ovoid, epithelioid, plasmacytoid, and/or clear cell features, containing a pale eosinophilic cytoplasm and ovoid or round nuclei without prominent nucleoli. Cells present with reticular, trabecular, or nested growth [291]. The tumor’s cellularity may vary with the presence and amount of a myxoid and/or myxohyaline stroma. In some cases, focal adipocytic metaplasia might be present. The majority of cutaneous myoepitheliomas show a typically lobular or multinodular architecture [290]. The lesion is usually well-circumscribed but unencapsulated, without connection to the overlying epidermis [296]. Tumors lack ductal differentiation, although entrapment of adnexal structures is common [290]. Immunohistochemically, variable expression of vimentin, cytokeratins, EMA, S100 protein, muscle actins, GFAP (glial fibrillary acidic protein), p63, and calponin is reported, with the lack of desmin positivity [287,296]. SOX10 seems to be a relatively reliable marker for staining cutaneous myoepitheliomas [297]. Differential diagnosis includes epithelioid benign fibrous histiocytoma, juvenile xanthogranuloma, melanocytic lesions, and less likely, epithelioid sarcoma for syncytial myoepithelioma [290].

Myoepithelial carcinoma, a malignant variant of cutaneous myoepithelioma, is characterized by severe cytological atypia with numerous mitoses [296]. Furthermore, in the absence of malignant cytomorphology and confirmed metastatic disease, infiltrative margins, satellite tumor nodules, tumor necrosis, and deep structures’ involvement are suggested to be ominous signs. However, criteria for malignancy in cutaneous myoepitheliomas have not been determined yet [287]. Nevertheless, malignancy carries a significant risk of distant metastases [290].

Myoepithelioma is considered a benign lesion; therefore, surgical excision with clear margins is performed (Table 2) [290,298]. The lesion may recur, although the metastatic potential is low [287,290]. Optimal treatment for myoepithelial carcinoma is unknown. WLE is performed, sometimes with sentinel lymph node biopsy. Complete resection during the local stage is suggested to provide long-term survival. The therapeutic approach in metastatic disease includes chemotherapy and radiotherapy, with poor outcomes [294,295].

## 3. Conclusions

Macroscopic features of each neoplasm are nonspecific, and they do not allow to diagnose the tumor without histopathological examination. Almost all lesions present as a slow-growing asymptomatic papule or nodule. Their surface is typically smooth; however, ulceration or crusting may occur, especially on poromas and porocarcinomas, apocrine carcinomas, and syringocystadenomas with syringocystadenocarcinomas papilliferum. Although their color might vary from white to blue-grey, the tumors are generally skin-colored, sometimes with erythema or telangiectasia. Lesions may occur in various parts of the body, where apocrine or eccrine glands are located [5,11,42,45,54,95,102,123,164,268,287].

Dermatoscopy may be helpful to distinguish adnexal tumors from other skin lesions. Typical features found in examination resemble the pattern observed in the case of other cutaneous lesions, such as the polymorphic vascular pattern seen not only in poroma or syringofibroadenoma papilliferum, but also in melanoma. Other nonspecific features include cyst or globules. Only leaf- and flower-like patterns seem unique for poroma [11,96,103,264,278].

A skin biopsy is essential to make a proper diagnosis. However, it is not easy because of the frequent use of superficial diagnostic biopsies, including shave and curettage procedures in this clinical setting [286,299]. The diagnostics should be performed in high-volume oncology centers.

Complete resection seems to be the optimal therapeutic approach for benign lesions [11,247,264,281,298]. Some of them may recur, although the metastatic potential is low and surgical treatment may be performed more than once [80,257,268,285,287,290]. Other therapeutic options include cryotherapy and CO_2_ laser therapy, photodynamic therapy, curettage, radiofrequency ablation, electrodesiccation and radiotherapy, as well as topical and systemic retinoid acids, 5-fluorouracil and imiquimod, with variable patient outcomes (Table 2) [206,213,228,231,235,236,241,242].

The therapeutic approach to malignancies is surgical intervention, including WLE, MMS, or amputation [2,54,86,101,118,119,144,145,162,169]. Sentinel node biopsy is sometimes performed, although its effectiveness is questionable, and more data seem to prove that it should not be widely recommended [3,120,144]. Laser ablation, cryotherapy, retinoic acid, trichloroacetic acid, CO_2_ laser, and radiotherapy can be performed only as additional treatment, and usually without distinction in outcome [3,72,73,74,75]. The role of adjuvant therapy remains unclear. Most tumors are resistant to radiotherapy and chemotherapy [11,54,57,80,88,97,294,300]. Primary or adjuvant radiotherapy may be considered in patients with microcystic adnexal carcinoma, and some cases of syringocystadenocarcinoma papilliferum, malignant mixed tumor, digital papillary adenocarcinoma, or signet-ring cell/histiocytoid carcinoma were also treated with successful radiation therapy [3,43,47,82,112,116,117,170,171,198]. Chemotherapy might be beneficial in patients with metastatic disease [87,151]. Anti-estrogenic treatment and HER2 inhibitors are used to treat hidradenocarcinoma, mucinous carcinoma, or apocrine adenocarcinoma, with variable outcomes [90,98,156]. Due to the long-term potential to recur and metastasize, regular follow-up and examination of the patient are recommended [19,80,92]. Only a few adnexal malignancies show a favorable prognosis, and no cases of recurrent or metastatic disease were reported [183,189,193,195].

**Table 1:** Genetic abnormalities reported in tumors derived from apocrine and eccrine glands.

Cancer Type	Genetic Abnormalities			Reference
	Mutation	Expression Downregulated	Expression Upregulated	
Adnexal adenocarcinoma NOS	ND			
Microcystic adnexal carcinoma	*TP53, JAK1, CDKN2A, CDKN2B, CDK11B, STAT3* In some cases, the authors have observed loss of function in cyclin-dependent kinase inhibitor 2A (*CDKN2A*), cyclin-dependent kinase inhibitor 2B (*CDKN2B*), *CDK11B* genes.	*CDKN2A, CDKN2B, CDK11B*	*p53, phospho-STAT3*	[39,48,301]
Porocarcinoma	*EGFR*, *HRAS*, *TP53*, *RB1*, *ATM*, *ARID1A*, *PIK3CA, CDKN2A,YAP1-MAML2, YAP1-NUTM1*	*TP53, RB1*	*EGFR, HRAS, YAP1-MAML2, YAP1-NUTM1*	[25,28]
Malignant neoplasms arising from spiradenoma, cylindroma, or spiradenocylindroma	*TP53, CYLD*	*CYLD, TP53*		[65,302]
Malignant mixed tumor	*PHF1-TFE3*			[78]
Hidradenocarcinoma	*ERBB2, TP53, t(11;19): CRTC1; MAML2, FGFR1, CDH1, MYST3, ZNF703, EGFR, PIK3CA, AKT-1*	*CDH1, TP53*	*FGFR1, MYST3, ZNF703*	[83,84,86,90,303]
Mucinous carcinoma	ND			
Endocrine mucin-producing sweat gland carcinoma	ND			
Digital papillary adenocarcinoma	*TP53, BRAF-V600E, FGFR2*	*TP53*	*FGFR2, BRAF-V600E*	[20,21,22]
Adenoid cystic carcinoma	*MYB-NFIB,MYBL1, SMARCA2*, *CREBBP,KDM6A, PIK3CA*			[126,129,130,304]
Apocrine carcinoma	*PIK3CA, KRAS.* In one case, mutations in *PIK3CA, KRAS* genes have been found.		*KRAS*	[158]
Squamoid eccrine ductal carcinoma	ND			
Syringocystadenocarcinoma papilliferum	ND			
Secretory carcinoma	*ETV6-NTRK3, NFIX-PKN1*			[173,175,176,177,178,179]
Cribriform carcinoma	ND			
Signet-ring cell/histiocytoid carcinoma	*NTRK3* *, CDKN1B, PIK3CA, ERBB2, CDH1*			[23,24,196].
Hidrocystoma/ cystadenoma	*MECT1-MAML2*			[305]
Syringoma	ND			
Poroma	*HRAS, ERBB4, APC*		*HRAS*	[306,307]
Syringofibroadenoma	ND			
Hidradenoma	*CRTC1-MAML2 PIK3CA, AKT1*			[244,307,308]
Spiradenoma	*CYLD, ALPK1*	*CYLD*		[250,254]
Cylindroma	*CYLD, MYB-NFIB,* *DNMT3A*	*CYLD*		[253,254]
Tubular adenoma	*BRAF, KRAS*		*BRAF, KRAS*	[263]
Syringocystadenoma papilliferum	*BRAF V600E, HRAS, KRAS*		*BRAF, HRAS, KRAS*	[273,274,275,276,277]
Mixed tumor	*PLAG1, EWSR1*			[26,27]
Myoepithelioma	*EWSR1* rearrangements (mostly *EWSR1-PBX3*)			[288,289,290,291]

**Table 2:** The multidisciplinary treatment cases reported for appendageal tumors.

Cancer Type	Surgery	Chemotherapy (Regiment, Dosing)	Reference	Radiotherapy (Gy)	Reference
Adnexal adenocarcinoma NOS	R	ND	[35]	ND	[35]
Microcystic adnexal carcinoma	R	NR	[3]	60–66 (2/fraction) or 45 (4.5/fraction)—NA or A of primary lesion	[3,47]
Porocarcinoma	R	NR	[11,57]	NR	[11,57]
Malignant neoplasms arising from spiradenoma, cylindroma, or spiradenocylindroma	R	A	[74,75]	A	[74,75]
Malignant mixed tumor	R	ND	[80,309]	LD	[80,82,309]
Hidradenocarcinoma	R	MD: 1. line: 5-fluorouracil, capecitabine 2. line: doxorubicin, platins, cyclophosphamide, vincristine, and bleomycin Targeted therapy (trastuzumab, tamoxifen)	[87,90,310]	A: 70 LR: 45–70	[47,88,89,310]
Mucinous carcinoma	R	NR	[92,97,98]	NR	[92,97]
Endocrine mucin-producing sweat gland carcinoma	R	ND	[101,109]	LD	[101,109,111]
Digital papillary adenocarcinoma	R	ND	[112]	LD	[112,116,117]
Adenoid cystic carcinoma	R	MD: 5-fluorouracil, cisplatin, adriamycin, cyclophosphamide, doxorubicin, vinorelbine	[139,140,141,142,311,312,313,314,315]	A	[311,314,316,317,318,319,320]
Apocrine carcinoma	R	NM: anthracyclines, taxanes, platinum drugs, cyclophosphamide, adriamycin, methotrexate, bleomycin HER2 inhibitors	[151,154,155,156,321]	A: 45–70	[151]
Squamoid eccrine ductal carcinoma	R	ND	[162,322]	ND	[161,162,322]
Syringocystadenocarcinoma papilliferum	R	LD	[170,172]	LD	[170,171,172]
Secretory carcinoma	R	ND	[183]	ND	[183]
Cribriform carcinoma	R	ND	[189,190,193]	ND	[189,193]
Signet-ring cell/histiocytoid carcinoma	R	NA, A—conventional and targeted therapy (HER2 inhibitors, tamoxifen, anti-androgen)	[23,197,199,204]	NA, A	[24,197,199,203,204]
Hidrocystoma/ cystadenoma	R	ND		ND	
Syringoma	R	ND		ND	
Poroma	R	ND		ND	
Syringofibroadenoma	R	ND		ND	
Hidradenoma	R	ND		ND	
Spiradenoma	R	ND		ND	
Cylindroma	R	ND		ND	
Tubular adenoma	R	ND		ND	
Syringocystadenoma papilliferum	R	ND		ND	
Mixed tumor	R	ND		ND	
Myoepithelioma	R	ND		ND	

R—recommended; NR—not recommended; ND—no data; NA—neoadjuvant therapy; A—adjuvant therapy; LR—local recurrence; NM—nodal metastasis; MD—metastatic disease; LD—limited data.

In a recent single-institution experience report, the median overall survival (OS) was 158 months (95% CI, 52–255). Age > 60 years was an unfavorable predictor of OS (HR 12.9, *p* < 0.0008) and recurrence-free survival (RFS) (HR 12.53, *p* < 0.0003). At the same time, nodal metastasis was a negative predictor of RFS (HR 2.37, *p* < 0.04) and disease-specific survival (HR 7.2, *p* < 0.03) [323].

In summary, neoplasms deriving from apocrine or eccrine glands are heterogeneous and uncommon skin lesions. Their pathogenesis remains unclear; however, genetic abnormalities, widely encountered in other neoplasms, are also found in cutaneous tumors with glandular origin (Figure 3, Table 1). Proper diagnosis is still challenging due to unspecific clinical presentation. Lesions require individual therapeutic management. Due to the rarity of these entities, limited data regarding molecular patterns or treatment options are available. Large trials concerning the comparison between various therapeutic approaches are certainly needed to optimize selecting the appropriate treatment and improving the overall prognosis.

## Data Availability

Raw pathology figures data was generated at Department of Pathology and Laboratory Diagnostics, Maria Sklodowska-Curie National Research Institute of Oncology and are available from the authors on request.
